# Insights Into Water Vapor Uptake by Dry Soils Using a Global Eddy Covariance Observation Network

**DOI:** 10.1111/gcb.70547

**Published:** 2025-10-13

**Authors:** Sinikka J. Paulus, Mirco Migliavacca, Markus Reichstein, Rene Orth, Sung‐Ching Lee, Arnaud Carrara, Anke Hildebrandt, Jacob A. Nelson

**Affiliations:** ^1^ Biogeochemical Integration, Max Planck Institute for Biogeochemistry Jena Germany; ^2^ Institute of Geoscience University of Jena Jena Germany; ^3^ Faculty of Environment and Natural Resources University of Freiburg Freiburg Germany; ^4^ European Commission, Joint Research Centre (JRC) Ispra Italy; ^5^ Fundacion Centro de Estudios Ambientales del Mediterráneo (CEAM) Paterna Valencia Spain; ^6^ Department Hydrosystemmodellierung Helmholtz Centre for Environmental Research—UFZ Leipzig Germany

**Keywords:** drylands, Eddy covariance, Fluxnet, land‐atmosphere exchange, negative latent heat fluxes, nighttime, non‐rainfall water input, soil hydraulic properties, soil water vapor adsorption

## Abstract

The exchange of water vapor between soil and atmosphere is a key component of land–atmosphere interactions, especially under dry conditions. Soil water vapor adsorption (SVA) occurs when the atmospheric water vapor pressure is greater than the soil air vapor pressure, which triggers the transport of water vapor from the atmosphere to the soil and its retention on the soil particle surface in liquid form. This process is largely caused by soil hydraulic properties and may play a significant role in dryland hydrology, yet remains understudied due to a lack of continuous, direct observations. In this study, we use globally distributed eddy covariance flux tower data to detect and characterize patterns of soil water vapor adsorption. We verify the consistency between negative latent heat fluxes as an indicator of water vapor movement toward the ground and the theoretical understanding of SVA. Our results reveal a relationship between the direction of the vapor gradient, as indicated by the direction of the latent heat flux, soil moisture, and near‐surface relative humidity, which is consistent with the understanding of a phase equilibrium at the pore scale of the soil. Distinguishing between random noise and physically explainable occurrences of negative latent heat fluxes enables the characterization of SVA occurrence in eddy covariance observations. SVA is detected most frequent in arid and semi‐arid regions, particularly in ecosystems with sparse vegetation such as savannas and dry shrublands. On average, SVA occurs for 4 ± 1.1 h per night, and may last up to 7 h in some locations. In certain sites, SVA occurs on more than 150 nights per year. These findings suggest that the eddy covariance method can help monitor SVA occurrence. Mapping the spatiotemporal patterns of SVA enhances our understanding of dryland land–atmosphere water fluxes and uncovers a previously overlooked component of the terrestrial water cycle.

## Graphical abstract

Using globally distributed flux tower data, we examined how soils adsorb water vapor from the atmosphere. We found that the direction of vapor exchange is closely linked to soil moisture and near‐surface humidity, consistent with soil–air equilibrium processes. The effect is most frequent in arid and semi‐arid ecosystems with limited vegetation, such as savannas and dry shrublands.

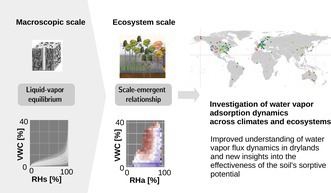

## 1 Introduction

The ability of the soil to retain liquid water is crucial for supporting nearly all terrestrial vegetation and microbial life. While rainfall serves as the primary source of liquid water for soils in most terrestrial environments, in regions with low annual rainfall or prolonged dry seasons, non‐rainfall water inputs—such as dew, fog and direct water vapor adsorption—can contribute a significant portion of the seasonal water budget (Kidron and Starinsky 2019; Paulus et al. 2022). This is particularly relevant in drylands, which are defined as regions where annual rainfall is less than 0.65 times the potential evapotranspiration, corresponding to an aridity index (AI) below 0.65 (Thomas et al. 1997). Drylands are commonly subdivided into four categories based on AI thresholds: hyper‐arid (AI < 0.05), arid (0.05 ≤ AI < 0.20), semi‐arid (0.20 ≤ AI < 0.50), and dry sub‐humid (0.50 ≤ AI < 0.65). Together, these regions comprise the largest terrestrial biome on Earth (∼60 million km^2^), covering over 40% of the global land surface (Safriel et al. 2005; Wang et al. 2022). Among non‐rainfall water inputs, dew and fog are widely recognized as important water sources for dryland ecosystems (Dawson and Goldsmith 2018; Agam and Berliner 2006; Wang et al. 2017; Binks et al. 2019). Soil water vapor adsorption (SVA), by contrast, remains relatively unknown and under‐researched, even within dryland studies (Agam and Berliner 2004; Saaltink et al. 2020; Kool et al. 2021).

However, SVA plays a relevant role for the daily and seasonal water balance in dryland ecosystems (Saaltink et al. 2020; Kool et al. 2021; Florentin and Agam 2017). SVA could even have an impact on the global carbon cycle, as it maintains thin films of soil moisture that influence carbonate chemistry. Recent studies suggest that it enables inorganic carbon uptake through CO_2_ dissolution and calcite precipitation in dryland soils, facilitating an observed uptake of −17 ± 15 g C m^−2^ per year by a dryland soil (Kim et al. 2024; Lopez‐Canfin et al. 2022a, 2022b). When extrapolated to the global extent of drylands, this mechanism could account for a significant proportion of the ‘missing’ terrestrial carbon sink (Kim et al. 2024). However, evaluating whether SVA is of such global relevance is difficult to assess due to the lack of long‐term, extensive spatial observations.

SVA is driven by the water vapor gradient between a wet atmosphere and comparatively drier soil air. This occurs predominantly in very dry soils, where the equilibrium pore air humidity is much lower than that in equilibrium with free water. It can therefore fall below that of the overlying atmosphere, generating vapor pressure gradients that drive fluxes from the atmosphere into the soil. This is caused by adsorption forces that depend on the specific surface properties of the soil and affect the soil pore vapor pressure at low soil water content (*VWC*, m^3^ m^−3^). In dryland ecosystems, strong diurnal temperature and humidity fluctuations drive alternating land–atmosphere vapor fluxes (Agam and Berliner 2006; Kool and Agam 2024; Lopez‐Canfin et al. 2022b). During the day, evaporation dominates due to the high energy input that heats the soil. This heating provides the energy needed to overcome cohesive forces between water molecules, allowing liquid water to transition into vapor and saturate the air‐filled soil pores. Meanwhile, the overlying atmosphere remains relatively unsaturated. As the energy input decreases in the evening, water molecules in the soil return to the liquid phase, leading to a decrease in vapor pressure within the soil pores. Once the atmospheric vapor pressure exceeds that of the soil pores, a gradient is established that drives water vapor from the air into the soil. There, they continue to adsorb at the soil particle surfaces, contributing to an increase in the soil's liquid water content (Verhoef et al. 2006).

Despite a good understanding of the process under equilibrated conditions at the soil sample scale—or, more precisely, the representative elementary volume scale (Hansen 1926; Edlefsen and Anderson 1943; Orchiston 1953; Tuller et al. 1999; Arthur et al. 2016), field observations remain sparse. Most observations of SVA originate from short‐term campaigns with a spatial extent of a few meters (Paulus et al. 2022). These studies show that SVA typically amounts to between 0.1 mm and 0.4 mm per night (Agam and Berliner 2004; Florentin and Agam 2017). Consequently, the process was considered to be neither hydrologically nor biologically relevant. However, these fluxes are significant compared to the diurnal evaporation rate typical of arid ecosystems. SVA has been shown to significantly contribute to diurnal evaporation dynamics (Qubaja et al. 2020; Paulus et al. 2022; Kool et al. 2021) and may help to fill gaps in our understanding of the seasonal water balance in dryland ecosystems (Katata et al. 2007).

Despite recent efforts to overcome technical limitations in measuring SVA at the field scale (Kool et al. 2021; Lopez‐Canfin et al. 2022b), current techniques remain restricted to the point scale, limiting their application across larger spatial areas. A promising alternative is the use of Eddy Covariance (EC) measurements, which capture the exchange of carbon, energy, and water vapor between ecosystems and the atmosphere, along with a series of biometeorological variables (Baldocchi et al. 2001; Baldocchi 2020). Global efforts to standardise EC‐based monitoring have led to global networks of EC data, such as FLUXNET (e.g., Pastorello et al. 2020), which have already significantly advanced our understanding of land–atmosphere interactions and ecosystem‐level processes (Baldocchi 2020; Migliavacca et al. 2021), supported model development (Jung et al. 2019; Nelson et al. 2024), and provided benchmarks for satellite product validation (Cescatti et al. 2012). However, the accuracy of EC‐derived water vapor fluxes is limited under nocturnal conditions due to low and intermittent turbulence (Mauder et al. 2013) and when atmospheric relative humidity (*RH*, %) is high (Zhang et al. 2023).

Recent studies have proposed that negative latent heat (*λE*, W $m^{-2}$) fluxes from EC, indicating water vapor movement towards the soil, can serve as an indicator of SVA (Florentin and Agam 2017; Paulus et al. 2024). Paulus et al. (2024) found that, during dry periods, 88% of detected negative *λE* flux events coincided with SVA detected by nearby weighing lysimeters. Although EC tends to underestimate the magnitude of SVA, the studies by Paulus et al. (2024) and Florentin and Agam (2017) independently suggest that EC data can provide a conservative yet consistent estimate of SVA occurrence. These findings highlight the need for a systematic test of whether SVA can be detected across ecosystems using the global EC network—a question that remains unexplored.

Here, we address two central research questions: (I) How likely is SVA to be detected in the fluxes in EC flux stations, and how can it be effectively distinguished from noise? (II) What is the global spatial distribution of SVA occurrence, and what environmental conditions favor its prevalence?

## 2 Material

### 2.1 Eddy Covariance Data

EC measurements were collected from 331 sites around the world as described in Nelson et al. (2024), with data available between 2001 and 2022. Each site's data originated from one of five different sources based on the most recent availability: FLUXNET2015 (Pastorello et al. 2020), ICOS Level2 data, ICOS Drought 2018 (Drought 2018 Team and ICOS Ecosystem Thematic Centre 2020), ICOS Warm Winter 2020 (Team and Centre 2022b), or the most recent AmeriFlux or ICOS releases as of December 2022. A list of sites used in this study, with the respective references, is given in Table 1. These datasets are processed consistently: Fluxes were calculated and submitted to the networks by the site teams before being post‐processed using the ONEFlux data processing pipeline (Nelson et al. 2024; Pastorello et al. 2020). This pipeline facilitates standardized gap‐filling methods across the datasets. *λE* flux measurements, along with their associated quality control (QC) flags, were used—restricted to good‐quality measured data (QC = 0 from ONEFlux). In addition, meteorological and soil data, including vapor pressure deficit (*VPD*, kPa), air temperature, volumetric soil water content (*VWC*, originally in $m^{3}m^{-3}$, hereafter expressed in vol.‐%), and rain were used to perform the analysis in this study. *VWC* data were quality‐checked and flagged following Jung et al. (2023), using multivariate consistency checks and outlier detection to exclude physically implausible values (e.g., *VWC* ≤ 0%). We converted *VPD* to atmospheric relative humidity (*RHa*, %) based on air temperature (*T*_*a*_, °C) using the Magnus equation with parameters from Sonntag (1990), as implemented in the *bigleaf* package (Knauer et al. 2018). The site's respective Plant Functional Type (PFT) classification according International Geosphere‐Biosphere Program (IGBP) land cover classification was reported by the PIs. There are a higher number of sites from temperate forests in North America and Europe (see Figure S1). We group the sites according to the PFT into the following land cover classes: Crops, Forests, Grasslands, Savannas, and Shrublands.

**TABLE 1:** Citation data for the 228 Eddy covariance sites used in this study.

Site	Citation	Site	Citation
AR‐SLu (Garcia et al. 2016)	AT‐Neu (Wohlfahrt et al. 2016)	AU‐Ade (Beringer and Hutley 2016b)	AU‐ASM (Cleverly and Eamus 2016b)
AU‐Cpr (Meyer et al. 2016)	AU‐DaP (Beringer and Hutley 2016a)	AU‐DaS (Beringer and Hutley 2016e)	AU‐Dry (Beringer and Hutley 2016d)
AU‐Emr (Schroder et al. 2016)	AU‐Gin (Macfarlane et al. 2016)	AU‐GWW (Pastorello et al. 2020)	AU‐How (Pastorello et al. 2020)
AU‐Lox (Liddell 2016)	AU‐RDF (Beringer and Hutley 2016c)	AU‐Rig (Pastorello et al. 2020)	AU‐Rob (Pastorello et al. 2020)
AU‐Stp (Pastorello et al. 2020)	AU‐TTE (Cleverly and Eamus 2016a)	AU‐Wac (Beringer, Hutley, et al. 2016)	AU‐Whr (Beringer, Cunningham, et al. 2016)
AU‐Wom (Arndt et al. 2016)	BE‐Bra (Warm Winter Team, and ICOS Ecosystem Thematic Centre 2022b)	BE‐Dor (Warm Winter Team, and ICOS Ecosystem Thematic Centre 2022b)	BE‐Lcr (Warm Winter Team, and ICOS Ecosystem Thematic Centre 2022b)
BE‐Lon (Warm Winter Team, and ICOS Ecosystem Thematic Centre 2022b)	BE‐Maa (Warm Winter Team, and ICOS Ecosystem Thematic Centre 2022b)	BE‐Vie (Warm Winter Team, and ICOS Ecosystem Thematic Centre 2022b)	BR‐CST (Antonino 2022)
BR‐Sa3 (Goulden 2016d)	CA‐Cbo (Staebler 2022)	CA‐Gro (McCaughey 2016)	CA‐LP1 (Black 2021)
CA‐NS1 (Goulden 2016f)	CA‐NS2 (Goulden 2016a)	CA‐NS3 (Goulden 2016b)	CA‐NS4 (Goulden 2016c)
CA‐NS5 (Goulden 2016h)	CA‐NS6 (Goulden 2016e)	CA‐NS7 (Goulden 2016g)	CA‐Oas (Black 2016)
CA‐Qfo (Margolis 2016)	CA‐SF1 (Amiro 2016b)	CA‐SF2 (Amiro 2016a)	CA‐SF3 (Amiro 2016c)
CA‐TP1 (M. A. Arain 2016b)	CA‐TP2 (M. A. Arain 2016a)	CA‐TP3 (M. Arain 2022b)	CA‐TP4 (M. A. Arain 2016c)
CA‐TPD (M. Arain 2022a)	CH‐Aws (Warm Winter Team, and ICOS Ecosystem Thematic Centre 2022b)	CH‐Cha (Warm Winter Team, and ICOS Ecosystem Thematic Centre 2022b)	CH‐Dav (Warm Winter Team, and ICOS Ecosystem Thematic Centre 2022b)
CH‐Fru (Warm Winter Team, and ICOS Ecosystem Thematic Centre 2022b)	CH‐Lae (Warm Winter Team, and ICOS Ecosystem Thematic Centre 2022b)	CH‐Oe1 (Ammann 2016)	CH‐Oe2 (Warm Winter Team, and ICOS Ecosystem Thematic Centre 2022b)
CN‐Cha (Zhang and Han 2016)	CN‐Cng (Dong 2016)	CN‐Dan (Shi et al. 2016)	CN‐Din (Zhou and Yan 2016)
CN‐Du2 (Chen 2016)	CN‐Du3 (Shao 2016b)	CN‐HaM (Tang et al. 2016)	CN‐Qia (Wang and Fu 2016)
CN‐Sw2 (Shao 2016a)	CZ‐BK1 (Warm Winter Team, and ICOS Ecosystem Thematic Centre 2022b)	CZ‐BK2 (Sigut et al. 2016)	CZ‐KrP (Warm Winter Team, and ICOS Ecosystem Thematic Centre 2022b)
CZ‐Lnz (Warm Winter Team, and ICOS Ecosystem Thematic Centre 2022b)	CZ‐RAJ (Warm Winter Team, and ICOS Ecosystem Thematic Centre 2022b)	CZ‐Stn (Warm Winter Team, and ICOS Ecosystem Thematic Centre 2022b)	DE‐Geb (Warm Winter Team, and ICOS Ecosystem Thematic Centre 2022b)
DE‐Gri (Warm Winter Team, and ICOS Ecosystem Thematic Centre 2022b)	DE‐Hai (Warm Winter Team, and ICOS Ecosystem Thematic Centre 2022b)	DE‐HoH (Warm Winter Team, and ICOS Ecosystem Thematic Centre 2022b)	DE‐Hzd (Warm Winter Team, and ICOS Ecosystem Thematic Centre 2022b)
DE‐Kli (Warm Winter Team, and ICOS Ecosystem Thematic Centre 2022b)	DE‐Lkb (Lindauer et al. 2016)	DE‐Lnf (Knohl et al. 2016)	DE‐Obe (Warm Winter Team, and ICOS Ecosystem Thematic Centre 2022b)
DE‐RuR (ICOS RI 2022)	DE‐RuS (Warm Winter Team, and ICOS Ecosystem Thematic Centre 2022b)	DE‐RuW (Warm Winter Team, and ICOS Ecosystem Thematic Centre 2022b)	DE‐Seh (Schneider and Schmidt 2016)
DE‐Tha (Warm Winter Team, and ICOS Ecosystem Thematic Centre 2022b)	DK‐Eng (Pilegaard and Ibrom 2016)	DK‐Fou (Olesen 2016)	DK‐Gds (ICOS RI 2022)
DK‐Sor (Warm Winter Team, and ICOS Ecosystem Thematic Centre 2022b)	DK‐Vng (Warm Winter Team, and ICOS Ecosystem Thematic Centre 2022a)	ES‐Abr (Warm Winter Team, and ICOS Ecosystem Thematic Centre 2022b)	ES‐Agu (Warm Winter Team, and ICOS Ecosystem Thematic Centre 2022b)
ES‐Amo (Poveda et al. 2016)	ES‐Cnd (Warm Winter Team, and ICOS Ecosystem Thematic Centre 2022b)	ES‐LgS (Reverter et al. 2016)	ES‐LJu (Warm Winter Team, and ICOS Ecosystem Thematic Centre 2022b)
ES‐LM1 (Warm Winter Team, and ICOS Ecosystem Thematic Centre 2022b)	ES‐LM2 (Warm Winter Team, and ICOS Ecosystem Thematic Centre 2022b)	FR‐Aur (Warm Winter Team, and ICOS Ecosystem Thematic Centre 2022b)	FR‐Bil (Warm Winter Team, and ICOS Ecosystem Thematic Centre 2022b)
FR‐EM2 (ICOS RI 2022)	FR‐Gri (Warm Winter Team, and ICOS Ecosystem Thematic Centre 2022b)	FR‐Hes (Warm Winter Team, and ICOS Ecosystem Thematic Centre 2022b)	FR‐Lam (Warm Winter Team, and ICOS Ecosystem Thematic Centre 2022b)
FR‐LBr (Berbigier and Loustau 2016)	FR‐Mej (Flechard et al. 2020)	FR‐Tou (ICOS RI 2022)	GF‐Guy (Warm Winter Team, and ICOS Ecosystem Thematic Centre 2022b)
GH‐Ank (Valentini, Nicolini, et al. 2016)	IL‐Yat (Warm Winter Team, and ICOS Ecosystem Thematic Centre 2022b)	IT‐BCi (Warm Winter Team, and ICOS Ecosystem Thematic Centre 2022b)	IT‐BFt (ICOS RI 2022)
IT‐CA1 (Sabbatini, Arriga, and Papale 2016)	IT‐CA2 (Sabbatini, Arriga, Gioli, and Papale 2016)	IT‐CA3 (Sabbatini, Arriga, Matteucci, and Papale 2016)	IT‐Col (Matteucci 2016)
IT‐Cp2 (Warm Winter Team, and ICOS Ecosystem Thematic Centre 2022b)	IT‐Cpz (Valentini, Dore, et al. 2016)	IT‐Isp (Gruening, Goded, Cescatti, and Pokorska 2016)	IT‐Lav (Warm Winter Team, and ICOS Ecosystem Thematic Centre 2022b)
IT‐Lsn (ICOS RI 2022)	IT‐MBo (Warm Winter Team, and ICOS Ecosystem Thematic Centre 2022b)	IT‐Noe (Spano et al. 2016)	IT‐PT1 (Manca and Goded 2016)
IT‐Ren (Warm Winter Team, and ICOS Ecosystem Thematic Centre 2022b)	IT‐Ro1 (Valentini, Tirone, et al. 2016)	IT‐Ro2 (Papale et al. 2016)	IT‐SR2 (Warm Winter Team, and ICOS Ecosystem Thematic Centre 2022b)
IT‐SRo (Gruening, Goded, Cescatti, Manca, and Seufert 2016)	IT‐Tor (Warm Winter Team, and ICOS Ecosystem Thematic Centre 2022b)	JP‐MBF (Kotani 2016b)	JP‐SMF (Kotani 2016a)
MX‐Tes (Yepez and Garatuza 2021)	MY‐PSO (Kosugi and Takanashi 2016)	NL‐Loo (Team and Centre 2020)	PA‐SPn (Wolf et al. 2016b)
PA‐SPs (Wolf et al. 2016a)	RU‐Fy2 (Warm Winter Team, and ICOS Ecosystem Thematic Centre 2022b)	RU‐Fyo (Warm Winter Team, and ICOS Ecosystem Thematic Centre 2022b)	RU‐Ha1 (Belelli et al. 2016)
SD‐Dem (Ardö et al. 2016)	SE‐Htm (Warm Winter Team, and ICOS Ecosystem Thematic Centre 2022b)	SE‐Lnn (Team and Centre 2020)	SN‐Dhr (Tagesson et al. 2016)
US‐A32 (Billesbach et al. 2022)	US‐AR1 (Billesbach et al. 2016b)	US‐AR2 (Billesbach et al. 2016a)	US‐ARb (Torn 2016b)
US‐ARc (Torn 2016a)	US‐ARM (Biraud et al. 2022)	US‐Bi1 (Rey‐Sanchez, Wang, Szutu, Shortt, et al. 2022)	US‐Bi2 (Rey‐Sanchez, Wang, Szutu, Hemes, et al. 2022)
US‐Blo (Goldstein 2016)	US‐CF1 (Huggins 2021)	US‐CF2 (Huggins 2022c)	US‐CF3 (Huggins 2022a)
US‐CF4 (Huggins 2022b)	US‐CRT (Chen and Chu 2016)	US‐CS2 (Desai 2022)	US‐GLE (Massman 2022)
US‐Goo (Meyers 2016b)	US‐Hn2 (Liu et al. 2022a)	US‐Hn3 (Liu et al. 2022b)	US‐HWB (Goslee 2022)
US‐IB2 (Matamala 2016)	US‐Jo1 (Vivoni and Perez‐Ruiz 2022)	US‐Jo2 (Vivoni and Perez‐Ruiz 2022)	US‐KFS (Brunsell 2022a)
US‐KLS (Brunsell 2022b)	US‐KS1 (Drake and Hinkle 2016a)	US‐KS2 (Drake and Hinkle 2016b)	US‐Lin (Fares 2016)
US‐LWW (Meyers 2016a)	US‐Me1 (Law 2016c)	US‐Me2 (Law 2022)	US‐Me3 (Law 2016a)
US‐Me4 (Law 2016e)	US‐Me5 (Law 2016d)	US‐Me6 (Law 2016b)	US‐MOz (Wood and Gu 2022)
US‐NC1 (Noormets et al. 2022)	US‐NR1 (Blanken et al. 2022)	US‐Oho (Chen et al. 2016)	US‐ONA (Silveira 2021)
US‐Rms (Flerchinger 2022b)	US‐Ro1 (Baker et al. 2022)	US‐Ro4 (Baker and Griffis 2022a)	US‐Ro5 (Baker and Griffis 2021)
US‐Ro6 (Baker and Griffis 2022b)	US‐Rws (Flerchinger 2022a)	US‐Snf (Kusak et al. 2022)	US‐SRC (Kurc 2022)
US‐SRG (Scott 2016a)	US‐SRM (Scott 2016b)	US‐Sta (Ewers and Pendall 2016)	US‐Syv (Desai 2016b)
US‐Ton (Baldocchi and Ma 2016)	US‐Tw2 (Sturtevant et al. 2022)	US‐Tw3 (Chamberlain et al. 2022)	US‐UMd (Gough et al. 2022)
US‐Var (Baldocchi et al. 2016)	US‐WCr (Desai 2016a)	US‐Whs (Scott 2016d)	US‐Wkg (Scott 2016c)
US‐xAE (NEON Network 2022)	US‐xBR (NEON Network 2022)	US‐xCL (NEON Network 2022)	US‐xCP (NEON Network 2022)
US‐xDC (NEON Network 2022)	US‐xDL (NEON Network 2022)	US‐xDS (NEON Network 2022)	US‐xGR (NEON Network 2022)
US‐xHA (NEON Network 2022)	US‐xJE (NEON Network 2022)	US‐xKA (NEON Network 2022)	US‐xKZ (NEON Network 2022)
US‐xMB (NEON Network 2022)	US‐xML (NEON Network 2022)	US‐xNG (NEON Network 2022)	US‐xNQ (NEON Network 2022)
US‐xRM (NEON Network 2022)	US‐xSB (NEON Network 2022)	US‐xSE (NEON Network 2022)	US‐xSR (NEON Network 2022)
US‐xST (NEON Network 2022)	US‐xTA (NEON Network 2022)	US‐xTR (NEON Network 2022)	US‐xUK (NEON Network 2022)
US‐xUN (NEON Network 2022)	US‐xYE (NEON Network 2022)	ZM‐Mon (Pastorello et al. 2020)	

From the 331 sites included in the dataset we excluded sites where (i) no *VWC* measurements were available, (ii) which were classified as wetlands or ice areas (iii) were located in high latitudes regions (Latitude ≥ 60 degree) as the latter two are associated with uncertainties in *VWC* sensor readings due to high soil organic carbon content and freezing thawing cycles. Finally, this study includes data from 228 sites. Table 1 lists all the included sites, along with the digital object identifier specific to each release. We selected nighttime half‐hourly data based on the nighttime classification provided in the dataset, which is typically based on a cross‐check of potential radiation and incoming solar radiation (Wutzler et al. 2018). We further excluded days with rain > 0 mm and half‐hours of < 5°C *T*_*a*_.

Note that in this study, we only evaluate flux directions, not flux magnitudes, as Paulus et al. (2024) found in a comparison with lysimeters that under dry conditions, EC underestimates SVA magnitudes by 25 to 47% but correctly detects SVA occurrence in 71% to 88% of negative nighttime fluxes. Applying a threshold filter based on friction velocity (u*) improved the detection by EC only by 1%. It, however, substantially decreased the amount of data for analysis. For this reason, we do not address energy balance non‐closure, *λE* flux magnitude uncertainty, friction‐velocity thresholds, or *λE* storage, all of which are relevant for assessing the uncertainties of the EC‐measured flux magnitude, especially at night (Padrón et al. 2020). We assume that these issues do not significantly and systematically affect the EC‐measured flux direction, as indicated by the results from Paulus et al. (2024).

### 2.2 Model Data and Remote Sensing Data

The soil texture was derived from SoilGrids250m version 2.0 (Poggio et al. 2021), a global data‐driven product compiled from national and local soil geographic databases to provide a globally consistent product. Only the top layer, from 0 to 5 cm below the surface, was used. Dominant clay mineral types within the top 0.3 m soil at each site were obtained from the global dataset by Ito and Wagai (2016), which provides mineralogical information at a 0.5° spatial resolution. Mineral types in this product are inferred based on the dominant soil taxonomy orders present within each grid cell (Ito and Wagai 2017). The Aridity Index (AI), defined as Precipitation/Potential Evapotranspiration, for the EC flux tower locations was taken from the Zomer et al. (2022) dataset, which is based on an annual average for the period 1970–2000, with the reference evapotranspiration calculated using the FAO Penman‐Monteith method (Allen et al. 1998). This is because many of the EC flux time series cover only 3 years and are therefore not suitable for calculating the mean climatic conditions. For climate classification, we followed the guidelines from the UN Environmental Programme (Thomas et al. 1997, see Table S1). To assess the coastline's influence on vapor availability, we calculated the distance to the nearest coast (GCL_FCS30.v1, 2020, Zhang, Zuo, et al. 2024; Zuo et al. 2025).

Since vegetation affects gas exchange between the soil and the atmosphere, we investigate the effect of average vegetation on SVA. To characterize maximum greenness at each EC site, we use the Near Infrared Vegetation Indices (NIRv, Badgley et al. (2017)) from the MODIS‐derived FluxnetEO product (Walther et al. 2022). We selected the Near‐Infrared Reflectance of terrestrial vegetation (NIRv) instead of the more widely used NDVI (Huete et al. 1997) since NDVI is more sensitive to soil reflectance and hence carries higher uncertainty in sparsely vegetated areas (Ding et al. 2016), where SVA can be expected. We used the portion of the FluxnetEO time series that overlaps with the flux tower measurements and calculated the 90th percentile of NIRv values to extract the maximum greenness. This approach is based on the assumption that the 90th percentile of NIRv values provides a robust estimate of peak vegetation activity, as it is less sensitive to noise and outliers commonly present in satellite‐derived time series. By limiting the analysis to the overlapping period, we aim to ensure temporal consistency between datasets and spatial consistency in the estimation of the greenness.

## 3 Methods

### 3.1 Conceptual Framework

Water vapor fluxes in the soil and across the soil‐atmosphere interface are driven by gradients in water potential defined by Fick's law, also known as vapor diffusion

(1) $$F=-\sigma_{\mathrm{eff}}\left(\Psi_{a}-\Psi_{s}\right)$$

where $\Psi_{a}$ is the water potential of the air above the soil, $\Psi_{s}$ is the water potential of the soil. $\Psi_{s}$ depends on *VWC* as described by soil water retention function. $\sigma_{\mathrm{eff}}$ represents an effective vapor transport parameter, accounting for tortuosity and soil‐air interactions. The sign of $\Psi_{a}-\Psi_{s}$ determines the direction of flux.

Water potential has a relative humidity equivalent (e.g., 100% *RH* = 0 MPa, and 95% *RH* = −6.8 MPa at 20°C) where relative humidity (*RH*, %) is derived from the Kelvin equation:

(2) $$\mathrm{RH}=\frac{e}{e_{0}\left(T\right)}=e^{\left(\frac{M_{w}\;\Psi }{\rho_{w}\;R\;T}\right)}$$

where RH represents the vapor pressure (*e*) relative to the saturation vapor pressure with respect to free water (*e*_0_). The term *M*_*w*_ is the molecular weight of water (0.018 kg mol^−1^), $\Psi_{s}$ is the soil water potential in kPa, *T* is temperature (Kelvin), *R* is the universal gas constant (8.31 J K^−1^ mol^−1^), and *ρ*_*w*_ is the density of water (kg m^−3^) (Or et al. 2022). Note that the effect of *T* on *RH* is small compared to the effect of $\Psi_{s}$ (and hence *VWC*) (Figure S2). Equation (2) is commonly used to describe the soil water retention curve in the dry moisture range under equilibrium conditions.

We can conceptually substitute the water potential terms for soil and atmosphere in Equation (1) with *RHs* and *RHa* using the Kelvin equation Equation (2).

(3) $$F=-\sigma_{\mathrm{eff}}\;\frac{\rho_{w}\mathrm{RT}}{M_{w}}\;\left(\ln\left({\mathrm{RH}}_{a}\right)-\ln\left({\mathrm{RH}}_{s}\right)\right)$$

In this formulation, vapor flux is expressed as a function of gradients in relative humidity rather than water potential. Although atmospheric relative humidity (*RHa*) is not defined by Equation (2), which applies strictly under thermodynamic equilibrium conditions in porous media, it can be conceptually interpreted as a proxy for atmospheric water potential (Binks et al. 2020).

### 3.2 Experimental Methodology

Several key variables from Equations (2) and (3), such as $\Psi_{s}$ or $\sigma_{\mathrm{eff}}$, are not routinely measured within the FLUXNET network. Estimating $\Psi_{s}$ from *VWC* using pedotransfer functions and gridded soil data is associated with considerable uncertainty (Weber et al. 2024). Notwithstanding these uncertainties, the strong dependence of the liquid–vapor equilibrium on soil water potential, as described in Equation (2), allows us to investigate its influence on the direction of water vapor fluxes at the ecosystem scale. We examine the relationship between *VWC* and *RHa* together with the direction of the *λE* flux. We argue that the observed curves relating *RHs* to *VWC* are comparatively close together, such that *VWC* carries a great deal of information about the vapor pressure of the air‐filled pore space in the soil.

Building on this argument, we hypothesize that *VWC* and *RHa* sufficiently reflect the underlying water potential gradient between the soil and the atmosphere to distinguish between two classes of gradient directions in *λE* fluxes at the ecosystem scale. These two classes are separated by a zero gradient line defined by the Kelvin equation Equation (2)—i.e., the soil‐specific relationship between *VWC* and *RHs*. Based on this assumption, we should find a relationship between observations of *VWC*, *RHa*, and the prevailing *λE* flux direction: (i) above the zero gradient line, *λE* fluxes should be positive (towards the atmosphere), and (ii) below the zero gradient line, *λE* fluxes should be negative (towards the ground). If our hypothesis is valid, then systematic negative *λE* fluxes can be distinguished from random noise in the EC measurements when interpreted in the context of *VWC* and *RHa*. Consequently, they can be attributed to SVA occurrence.

In order to distinguish conceptually between the scales of observations (soil vs. ecosystem), we introduce the concept of the *apparent Ecosystem Vapor Equilibrium* (EVEa) as the emergent functional relationship between *VWC*, *RHa*, and *λE* flux direction at the ecosystem scale (i.e., scale emergent property). While conceptually related to the processes described by Equations (1) and (2), this concept integrates commonly measured variables in the soil and the atmosphere, without requiring assumptions about detailed hydraulic properties of the soil that would be necessary to solve these equations literally.

**FIGURE 1:**
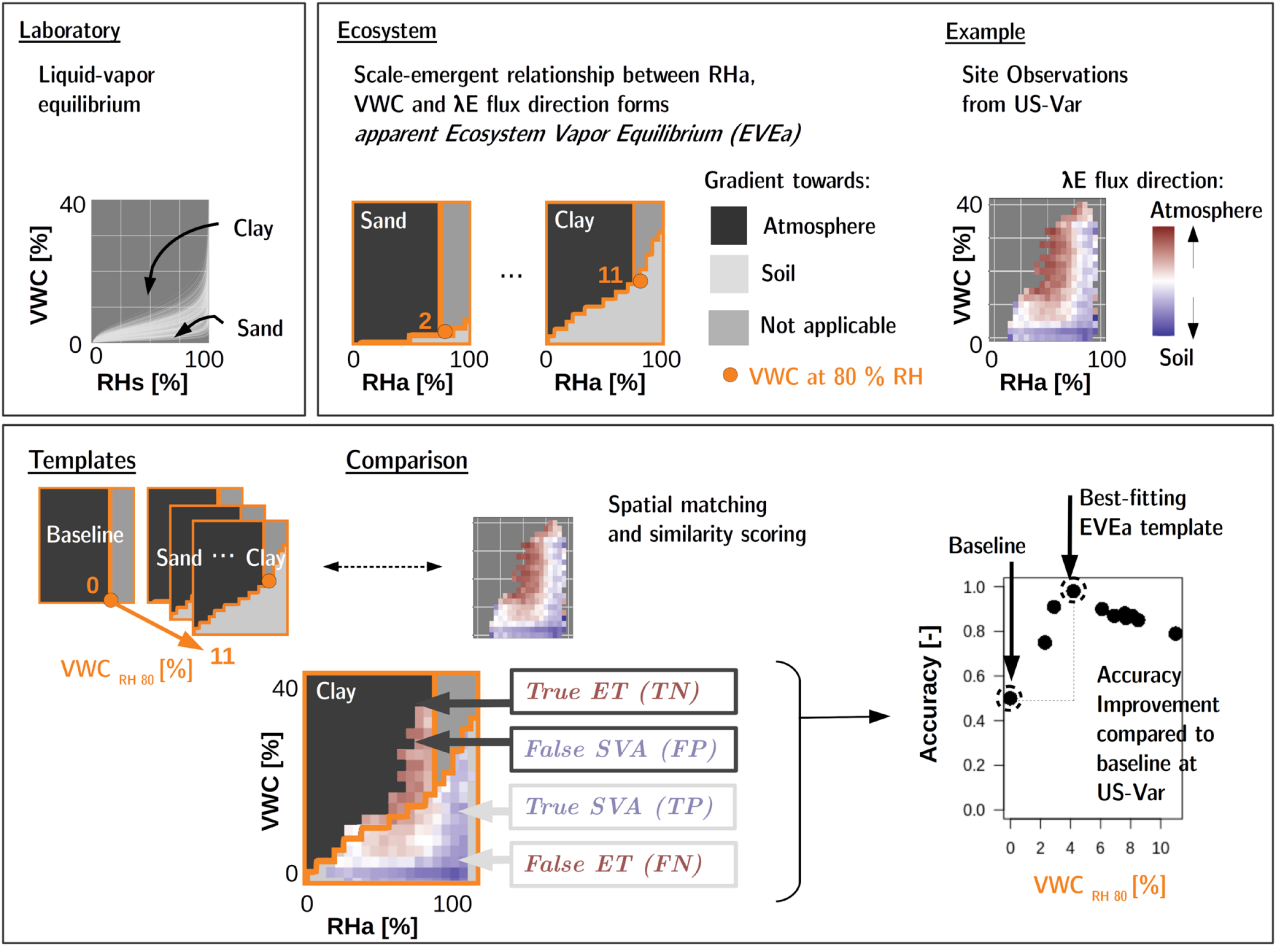
Methodological framework for identifying ecosystem‐scale apparent vapor equilibrium (EVEa). Laboratory‐derived liquid–vapor equilibrium (left) provides the basis for hypothesizing an emergent ecosystem relationship between relative humidity at air–soil interface (RHa), volumetric water content (VWC), and latent heat flux direction (*λE*), reflecting the underlying gradients in vapor pressure (center). Site observations (right) are compared with texture‐specific EVEa templates via spatial similarity scoring. Observed and predicted flux directions are classified as true/false positives and negatives, enabling calculation of accuracy. The best‐fitting EVEa template is selected based on accuracy improvement over a baseline (bottom right).

Figure 1 illustrates our methodology. We derive the theoretical shapes of the boundary between the gradient directions, the EVEa, based on publicly available soil hydraulic measurements in the dry range of the soil water retention curve from 542 soil samples (Hohenbrink et al. 2023). These are predominantly not from FLUXNET sites, but cover a wide range of soil textures including soils with up to 65% clay and 93% silt, and 100% sand (Figure S3) (Hohenbrink et al. 2023). The measurements were performed in the laboratory using a dew‐point potentiometer (WP4C, METER Group Inc., USA). The fitted soil water retention model interpolating between measurements is the Peters‐Durner‐Iden model (Peters et al. 2021, 2023; Hohenbrink et al. 2023). The Peters‐Durner‐Iden model specifically accounts for non‐capillary water in thin films on particle surfaces, resulting in more realistic retention at dry moisture values compared to the widely used Van‐Genuchten‐Mualem Model (Peters et al. 2023). As illustrated in Figure S3, the relationship between *VWC* and *RHs* varies with clay content. We therefore calculate the average relationship for the different USDA soil textures (*n* = 11). These serve as a benchmark for expected texture‐specific behavioural differences of the EVEa. We name the EVEa based on the *VWC* in equilibrium with air at 80% *RHs*, denoted as *VWC*_*RH*80_, which corresponds approximately to a water potential of −300,000 hPa (Figure S4).

Our null hypothesis states that *λE* flux direction is independent of *VWC* and *RHa*. Under this assumption, observations would either represent random noise (no discernible dominant flux direction) or exhibit a single prevailing direction, namely an upward flux toward the atmosphere, consistent with conventional interpretations of EC *λE* fluxes.

To test our hypothesis with real observations, we turn to the FLUXNET dataset and analyze how *λE* flux direction varies with *VWC* and *RHa*. For each FLUXNET site, we determine the dominant *λE* flux direction across the locally observed ranges of *VWC* and *RHa* by grouping half‐hourly observations into bins of 5% for *RHa* and 2% for *VWC*. Within each bin, we then calculate the relative frequency of negative *λE* fluxes (negative *λE*/all *λE*). We classify the bins into: dominantly towards the atmosphere, when the fraction is < 0.45, dominantly towards the soil when the fraction is >0.55. Fractions between ≥0.45 and ≤0.55 are interpreted as random noise, assuming a symmetric distribution of the random error (Lasslop et al. 2008; Richardson et al. 2008; Vitale et al. 2019). We only use good‐quality measured data (QC = 0 from ONEFlux), and each bin must contain at least 20 observations, corresponding to approximately 10 h of measurements under the respective conditions. This threshold ensures that each bin contains a sufficient sample size from which a meaningful dominant flux direction can be inferred. We limit this part of our analysis to nighttime data. This decision is motivated by the heteroscedastic nature of random errors in EC flux measurements, where the error magnitude increases with flux magnitude (Lasslop et al. 2008). In our dataset, *λE* fluxes during the day are generally dominated by transpiration, even when the upper soil layers are dry, as vegetation often has access to deeper soil moisture. Since *λE* fluxes are generally low at night, the exclusive use of nighttime data ensures that random noise is similarly high and symmetrical for both positive and negative flux directions. This improves the comparability of the derived flux direction distributions.

Having established the observed flux‐direction patterns, the next step is to evaluate whether these observations are consistent with our hypothesis. To do so, we apply standard classification metrics (Lever et al. 2016). For each site and texture‐specific EVEa template, we calculate accuracy (ACC), true positive fraction (TPF), false positive fraction (FPF), and true negative fraction (TNF). TPF represents correctly identified SVA events, while FPF indicates cases where negative *λE* fluxes occur under conditions where SVA is not possible. An overview of these metrics is provided in Section S6. We select the best‐fitting EVEa based on the highest accuracy; if this corresponds to EVEa ${\mathrm{VWC}}_{\mathrm{RH}\;80}=0$, SVA is either absent or undetectable at the site.

We exclude conditions from flux direction analysis where SVA is unlikely due to high *VWC* but where negative *λE* fluxes may still occur due to dew formation or fog deposition. Based on findings from ecosystem‐level studies (Ritter et al. 2019), we use a *RHa* threshold of 75%, above which dew formation is likely. Therefore, flux directions are not accounted for in the classification metrics under conditions exceeding this *RHa* threshold when *VWC* is also too high for SVA, as defined by our reference laboratory dataset (Hohenbrink et al. 2023).

## 4 Results

### 4.1 Patterns and Temporal Dynamics of Latent Heat Flux Directions

**FIGURE 2:**
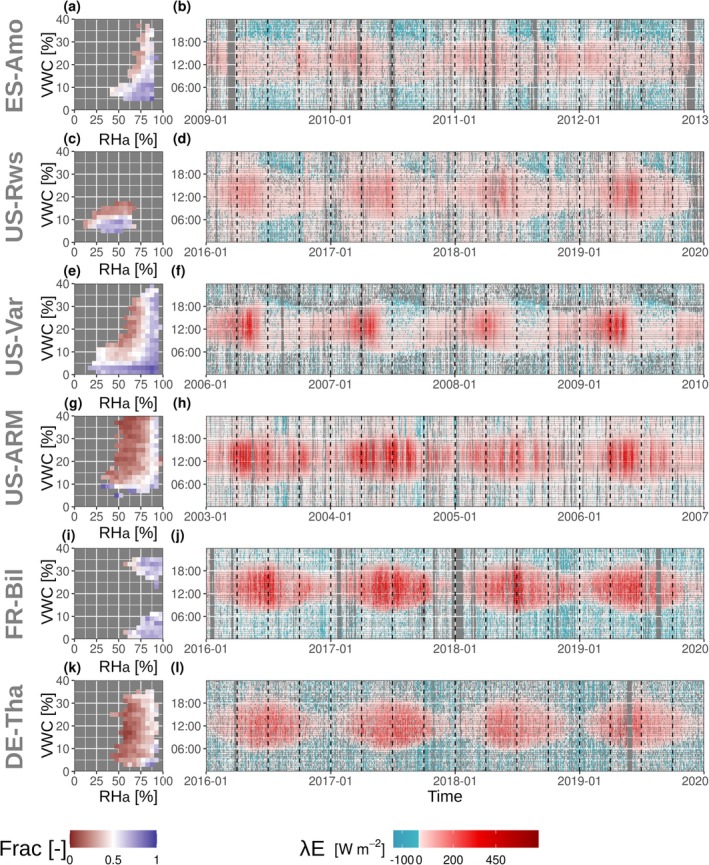
Patterns in latent heat (*λE*) flux direction at six Eddy Covariance (EC) sites. *Left column*: Fraction of negative *λE* fluxes relative to total measured fluxes under different conditions of atmospheric relative humidity (*RHa*) and volumetric water content (*VWC*). *Right column*: Dynamics of *λE* flux magnitudes at the diel and seasonal scales over 3 years. The dashed vertical lines divide the measurements into periods of 3 months. Ancillary information for each site is provided in Table 2.

Our results show that the observed dominant flux direction of *λE* varies with prevailing *RHa* and *VWC* conditions (examples shown in the *left column* of Figure 2, ancillary information given in Table 2). Specifically, under low *RHa* and high *VWC*, *λE* fluxes are predominantly positive. Under high *RHa*, negative *λE* fluxes dominate regardless of *VWC*. However, when *RHa* is below 75%, negative *λE* fluxes occur exclusively when *VWC* is less than ∼15%. This pattern is evident at semi‐arid and arid sites (e.g., ES‐Amo, US‐Rws, US‐Var, US‐ARM). Distinct patterns emerge among the temperate forest sites. At FR‐Bil, negative *λE* fluxes consistently occur under conditions of high *RHa*. At DE‐Tha, *λE* remains positive even at similarly high *RHa* levels as FR‐Bil and other dryland sites. Note that the apparent gap in *VWC* values for site FR‐Bil (Figure 2i) results from the sandy soil's rapid drying behavior and the exclusion of bins with fewer than 20 half‐hourly observations.

**TABLE 2:** Ancillary information for the sites shown in Figure 2.

Site	PFT	Aridity	Acc at 0	Max acc.	*VWC*_*RH80*_	TP	Median *VWC*	Dist. coast
			[−]	[−]	[%]	[−]	[%]	[km]
ES‐Amo	OSH	Arid	0.500	0.961	8.08	34	15.1	4
US‐Rws	OSH	Semi‐arid	0.582	0.855	11.0	23	15.7	688
US‐Var	GRA	Semi‐arid	0.619	0.972	4.20	45	10.0	136
US‐ARM	CRO	Semi‐arid	0.902	0.944	4.20	10	26.5	908
FR‐Bil	ENF	Humid	0.292	0.927	11.0	33	16.9	19
DE‐Tha	ENF	Humid	0.938	0.951	2.31	5	18.5	328

Abbreviations: Acc, accuracy; PFT, plant functional type; TP, true positives; *VWC*, volumetric soil moisture content; *VWC*_*RH80*_, apparent ecosystem vapor equilibrium.

Temporal differences in the dynamics of *λE* flux direction are also evident (*right column* in Figure 2). In arid and semi‐arid sites, negative *λE* fluxes occur predominantly on a seasonal basis, mainly following the sharpest decline in evapotranspiration (US‐Rws, US‐Var). An exception is ES‐Amo, an arid shrubland site, where negative *λE* fluxes persist year‐round. This may be due to a combination of the site's extreme aridity, its proximity to the coast (just 4 km from the shoreline), and its clay mineralogical composition that likely favors SVA. The topsoil contains 23.7% Illite/Mica and 13.5% Smectite, while the subsoil is estimated to have up to 76.3% Illite/Mica, based on Ito and Wagai (2016). These minerals, particularly Smectite, are known to have a large specific surface area to which vapor can adsorb and may therefore amplify the SVA signal observed at the ecosystem scale.

In contrast to the arid climates, wet climates exhibit negative *λE* fluxes throughout the year, with flux directions fluctuating between positive and negative at night, particularly at DE‐Tha.

### 4.2 Validity of Flux Direction Hypotheses

**FIGURE 3:**
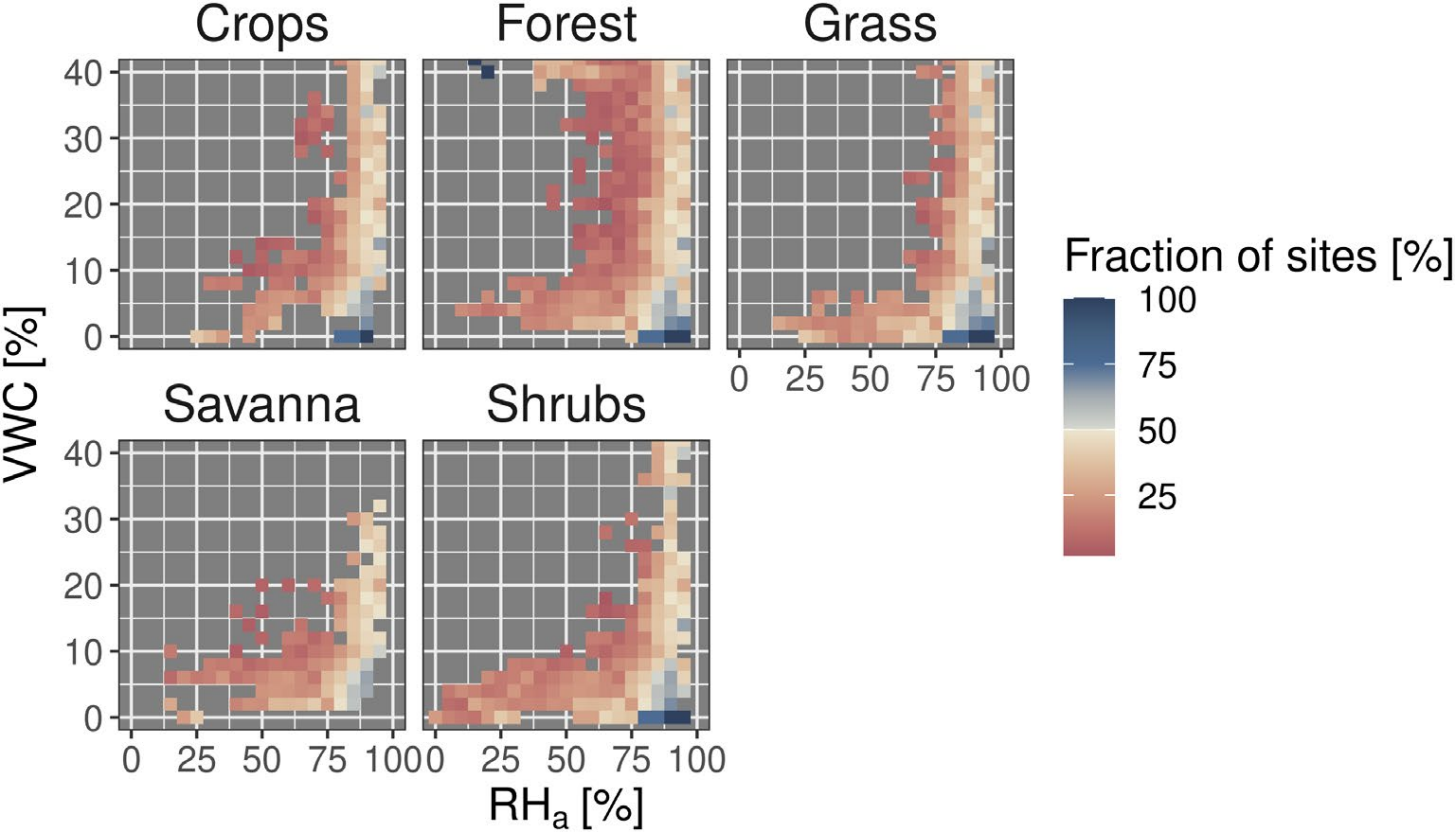
Prevalence of negative latent heat fluxes across EC sites under varying soil moisture (*VWC*) and atmospheric relative humidity (*RHa*) conditions for different vegetation types. The colorbar is split at 50% to emphasize conditions where the majority of sites detect predominantly negative *λE* flux direction.

Figure 3 illustrates the prevalence of negative *λE* fluxes across all EC sites under varying *VWC* and *RHa* conditions for different vegetation types. Across sites and vegetation types, negative *λE* fluxes dominate in a narrow range of conditions. Namely, they dominate at high *RHa* conditions independent of *VWC*. But for *RHa* values lower than 75%, they only dominate when *VWC* is less than ∼10%. This relationship emerges across all vegetation types. For croplands, there is, however, less data available in the range of low *VWC*. Compared to other vegetation types, forests behave differently in that we observe negative *λE* fluxes substantially more often, also at lower *RHa* during high *VWC*.

To evaluate the hypothesis that there is a detectable empirical relationship between *VWC*, *RHa*, and the dominant *λE* flux direction, shaped by the underlying hydraulic properties, we assess whether the EVEa can be used to correctly classify EC observations into the two dominant flux directions.

**FIGURE 4:**
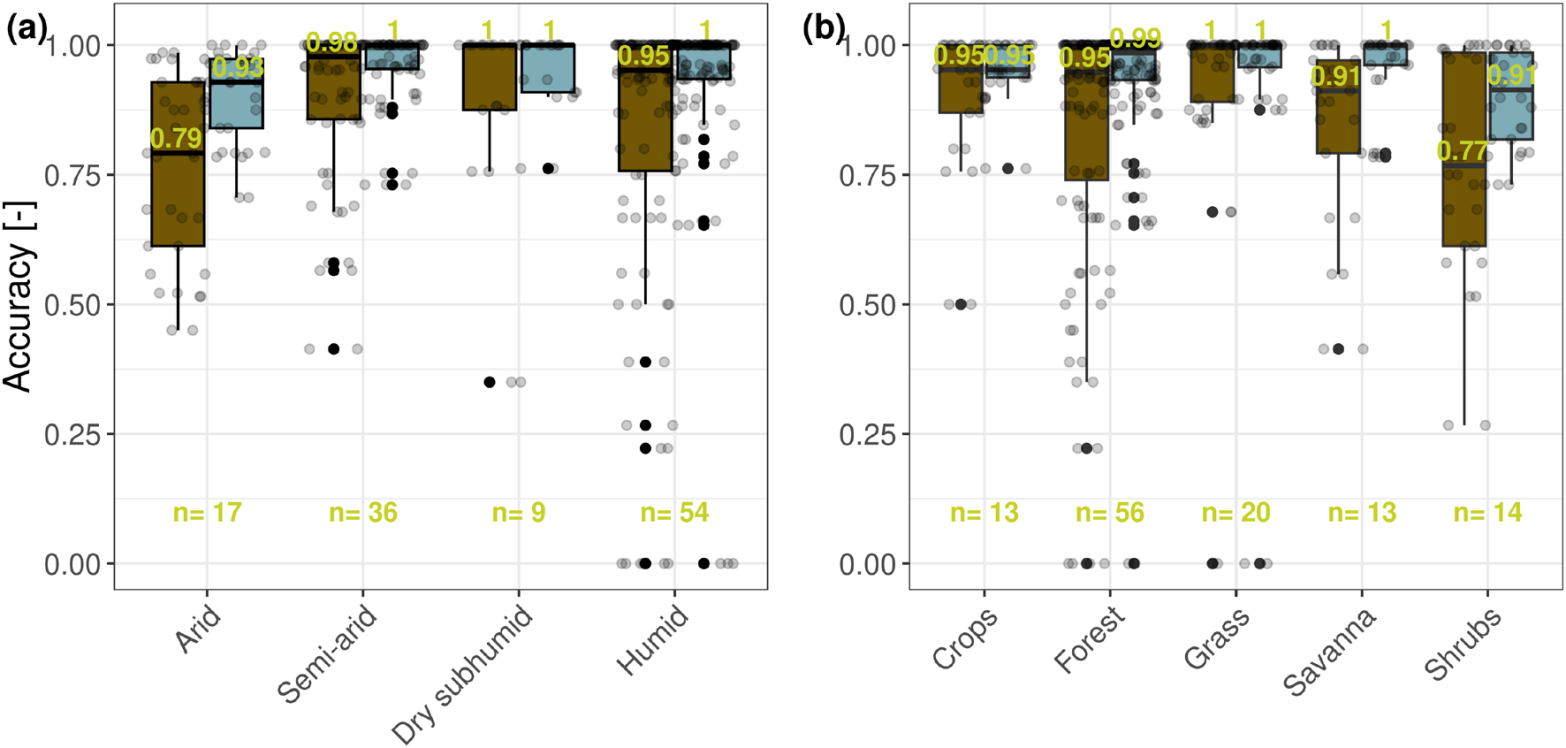
Accuracy of latent heat flux direction classification for the baseline (null hypothesis) (brown) and the best‐fitting apparent Ecosystem Vapor Equilibrium (EVEa) template (light blue), shown as boxplots for (a) levels of aridity and (b) different plant functional types. The results are obtained based on the methodology illustrated in Figure 1. The dots show the accuracy of the individual sites within each of the groups. The median accuracy and the number of sites per group are indicated by the numbers at the top and bottom of the boxplots, respectively.

The results are shown as accuracy improvements for different climates and PFTs in Figure 4. We find the strongest improvement in accuracy for sites in arid climates (from 0.79 to 0.93) and for shrublands (from 0.77 to 0.93). Individual sites across PFTs and levels of aridity also show substantial improvement.

The improvement in accuracy is mainly caused by an improvement of the TPF, while the FPF (TNF) is low (high) across the tested templates of the EVEa (Figure S5). These results confirm that negative *λE* fluxes seldom dominate when *VWC* is high and *RHa* is low. Sites with an improvement of accuracy of minimum 3% are individually shown in Figure S6. Notably, sites with many TPs—meaning they have many observations under conditions of low *VWC*– show an accuracy increase from moderate (ACC~0.6) to high (ACC > 0.9). However, some individual sites with few TPs also show substantial accuracy gains, which are more likely influenced by artifacts. In summary, interpreting negative *λE* fluxes as signals of the underlying phase equilibrium increases classification accuracy by about 15% on average, across drylands and shrublands, and by more than 30% at certain sites with favorable conditions.

We therefore next examine whether these accuracy improvements align with site‐specific soil properties that are expected to influence vapor adsorption. In theory, the highest accuracy should be obtained with the EVEa template that reflects the site's clay content and clay mineralogy. However, we did not find a significant linear relationship between clay content and the selected EVEa template, even when accounting for climate and PFTs. In contrast, a significant negative relationship was observed between the EVEa template and sand content at semi‐arid sites and shrubland sites (see Figure S7), aligning with expectations that sandy soils exhibit reduced vapor adsorption compared to clay‐rich soils. The lack of correlation with clay may be due to higher measurement uncertainty, and because certain clay types—such as kaolinite—can behave similarly to sand with respect to vapor adsorption.

We also tested whether the expected relationship between clay mineral type and the best‐fitting EVEa template was reflected in the FLUXNET observations—specifically, whether sites with smectite showed higher EVEa values than those with illite/mica or kaolinite (i.e., Smectite > Illite/Mica > Kaolinite). However, this pattern was not supported by the observations (Figure S8). Variability within each mineral group was high, and no consistent ranking of EVEa values by mineral type emerged across sites, even when accounting for climatic differences.

Figure S7 further illustrates that there is no relationship between the EVEa template and the maximum accuracy gained at each site.

### 4.3 Inter‐Annual Frequency and Duration of Soil Water Vapor Adsorption

**FIGURE 5:**
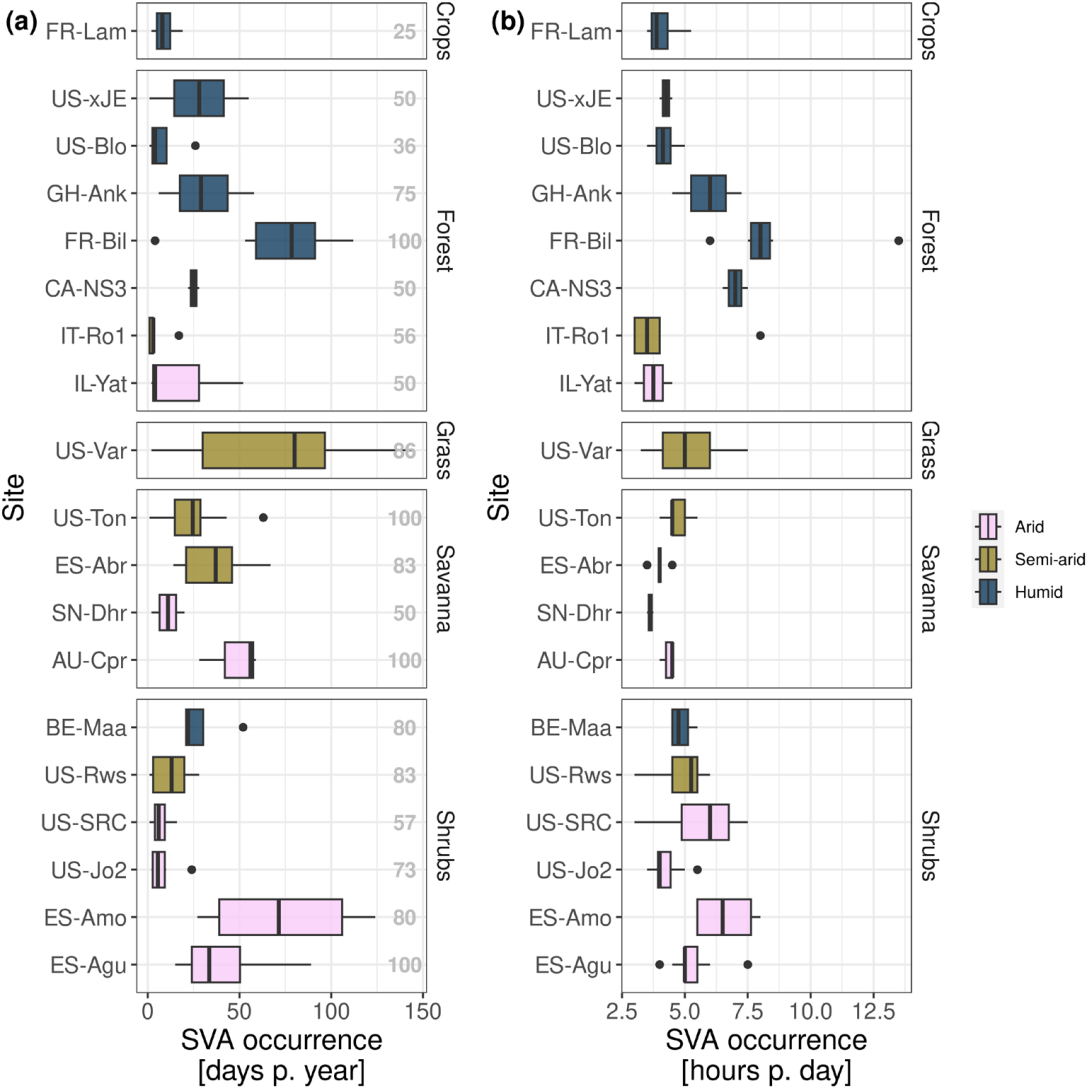
Median occurrence of soil water vapor adsorption (SVA) in days per year (a) and hours per day (b) based on eddy covariance measurements across different PFTs, colored by aridity class. Medians are calculated from years with observations under the specific climate conditions enabling SVA. Only sites with at least 1 year exhibiting ≥ 10 days with ≥ 3 h of SVA are included. The grey numbers in field (a) show the percentage of years in which SVA was detected at the respective site.

Having established that interpreting negative *λE* fluxes through the lens of soil‐atmosphere vapor gradients improves classification accuracy, we now use this framework to distinguish systematic SVA signals from random noise. In doing so, the classification system becomes a diagnostic tool for interpreting fluxes. Specifically, we analyzed the time series data from the EC sites to characterize the occurrence frequency of SVA within years, by summing the half‐hour records where a negative *λE* was detected under conditions of low *VWC* (TP). For each site, we used the EVEa that yielded the highest accuracy. Figure 5 presents the median number of days per year with SVA, including only those sites where at least 1 year exhibited ≥ 10 days with ≥ 3 h of SVA, to focus the analysis on locations where the signal is more consistently detectable and less likely to reflect isolated or uncertain events. Unlike other parts of this study, here we utilize the full 24 h of high‐quality *λE* flux measurements.

The EC method detected the highest frequency of SVA at the US‐Var grassland site, with a median of 80 days per year. However, this was the only grassland where substantial SVA fluxes were detected. Among the other PFTs, arid and semi‐arid savannas showed the highest SVA occurrence (median of 31 days per year), followed by forests (25 days), shrublands (18 days), and croplands (8 days). Notably, some individual sites exhibited much higher median annual SVA frequencies regardless of PFT or aridity class, including FR‐Bil (humid forest, 79 days), ES‐Amo (arid shrubland, 71 days), and AU‐Cpr (arid savanna, 56 days).

In addition, interannual variability was considerable—for instance, US‐Var exhibited an interquartile range of 66 days per year, with values ranging from 0 to 145 days over the 22‐year period. When present, SVA events generally lasted fewer than 7 h per day, with the exception of FR‐Bil, which had a median duration of 8 h. Exceptionally high SVA occurrence frequency at individual sites can be partly explained by their proximity to the ocean (e.g., FR‐Bil, ES‐Amo, ES‐Agu, GH‐Ank) and/or the presence of dominant clay mineral groups with high specific surface area (e.g., AU‐Cpr, FR‐Bil, US‐Var, ES‐Amo) (see Figure S9). However, no single factor consistently explains the observed patterns across all sites. Due to the high level of correlation, it is also not possible to fully disentangle the effects of aridity and plant cover (NIRv, see Figure S10). Given the limited availability of site‐specific information on key soil properties (e.g., texture, mineralogy), experimental setup (e.g., sensor placement), and microclimatic conditions (e.g., surface roughness), we refrain from attributing observed patterns to individual drivers without more detailed, site‐level data.

### 4.4 Uncertainty in Soil Water Vapor Adsorption Detection

**FIGURE 6:**
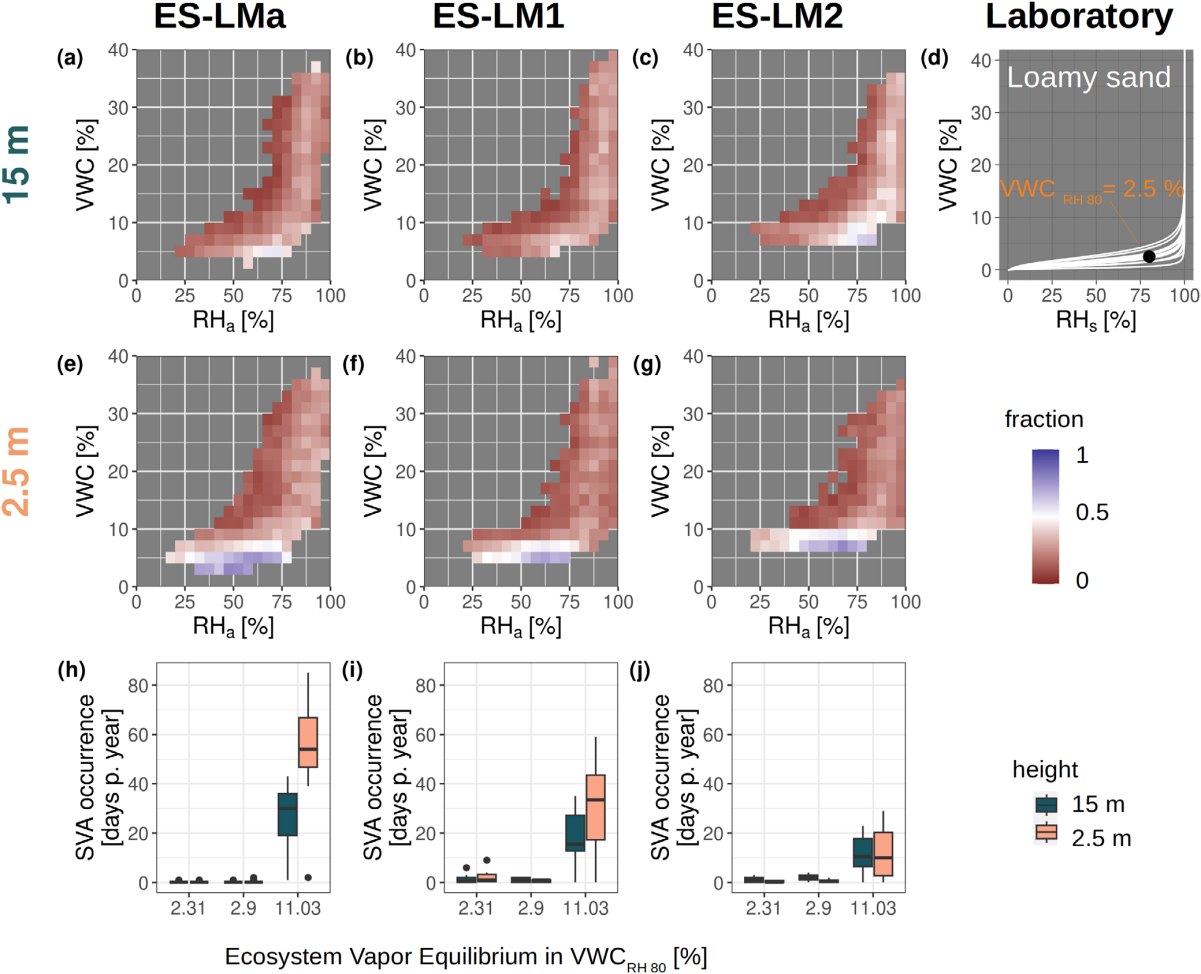
Fractions of negative latent heat fluxes relative to total measured fluxes, shown as a function of atmospheric relative humidity (*RHa*) and volumetric water content (*VWC*) for three colocated sites: ES‐LMa, ES‐LM1, and ES‐LM2. Panels (a–c) display observations from 15 m height, while panels (e–g) show measurements from 2.5 m height. Panels (h, l, and j) depict the annual frequency of soil water vapor adsorption (SVA) events, based on different apparent Ecosystem Vapor Equilibrium (EVEa) templates. The soil sample scale‐measured equilibrium relationship between soil relative humidity and *VWC* is presented in panel (d).

Figure 6 presents measurements from three co‐located sites in the Majadas de Tiétar experimental setup. Each site is equipped with two EC systems: one on a tall tower (15 m) and one on a short tower (1.6 m). This design allows us to assess how tower height and small‐scale ecosystem variability influence the detection of SVA patterns.

The results show a clear height‐dependent effect: the tall towers (panels a–c) do not detect predominant negative *λE* fluxes under dry conditions, whereas the short towers do. This difference significantly affects the interpretation of SVA detection frequency (panels h, f, j). At this site, laboratory measurements of soil texture and water retention characteristics in the dry range from 20 topsoil samples are available (panel d). These data enable a comparison between ecosystem measurements and independently measured laboratory values. Using the EVEa template that best matches the site's soil texture (loamy sand), we estimate a median SVA occurrence of just 0 to 5 days per year. However, applying the EVEa template corresponding to pure clay (${\mathrm{VWC}}_{\mathrm{RH},80}$ = 11.03), which yields the highest event inclusion, substantially increases the estimated SVA frequency across sites and tower heights. The subcanopy tower at ES‐LMa detects the highest frequency, with up to 84 nights per year, while other towers show a median of approximately 35 nights per year.

For context, Paulus et al. (2024) reported a 75% recall rate for the Majadas subcanopy tower, relative to automated weighing lysimeter observations, with a mean event frequency of 112 nights per year. By comparison, the current methodology detects only 54 nights per year over the same period. This suggests that even when using an EVEa template corresponding to pure clay—representing the highest possible separation area between true SVA events and random noise—we still obtain only a conservative estimate of the actual SVA frequency observed at this site. Factors such as tower height, vegetation type, aridity, soil–atmosphere coupling, and local soil hydraulic properties likely influence SVA detection from EC differently across sites. Unraveling their relative contributions remains an important open question for future research and is discussed in more detail in the following section.

## 5 Discussion

### 5.1 Hotspots of Soil Water Vapor Adsorption Across Sites

Most previous studies investigating spatial variation in SVA have been limited to plot‐ or catchment‐scale observations spanning a few meters to kilometers (Kosmas et al. 2001; Kool et al. 2021; Verhoef et al. 2006). Studies comparing SVA across climatically and ecologically diverse ecosystems are still lacking. Here, we address this gap by leveraging a globally distributed EC dataset to identify consistent patterns of SVA occurrence across a wide range of climates and vegetation types.

Our analysis reveals a general tendency for arid and semi‐arid ecosystems to exhibit the strongest SVA signals, both in terms of annual frequency and daily duration. Within the FLUXNET network, we identify multiple “hotspots” of SVA activity, particularly at semi‐arid sites with sparse vegetation, such as US‐Var, ES‐Amo, ES‐Abr, ES‐Agu, and AU‐Cpr. SVA is also consistently detected at several U.S. rangelands (US‐SRC, US‐SRM, and US‐Jo2). Notably, some forested sites, such as FR‐Bil, IL‐Yat, GH‐Ank, and US‐xJE—also exhibit significant SVA signals.

Several factors may explain the high frequency of SVA observed at specific sites. Proximity to the ocean likely plays a role, as seen at FR‐Bil (18.6 km) and GH‐Ank (28.2 km), where atmospheric moisture availability is elevated. High content of clay mineralogy with large specific surface area could also contribute, such as the 34.1% estimated topsoil smectite content at AU‐Cpr and 28.6% illite/mica at US‐xJE. In addition, site‐specific land management and vegetation characteristics may influence SVA. For instance, US‐xJE, a humid forest site, undergoes regular prescribed burns, which may alter surface conditions and promote adsorption. US‐Blo's nighttime EC footprint is not dominated by the forest canopy but by adjacent sparse plantations, potentially affecting flux measurements. FR‐Bil is a young pine plantation with a short canopy and recent thinning, located on sandy soils that drain rapidly—conditions that may favor SVA events during summer. Overall, no single factor appears to universally explain high SVA frequency. Rather, site‐level variation seems driven by a combination of climatic, soil, vegetation, and management factors.

Nevertheless, Figure S11 showcases selected EC sites with a high frequency of identified SVA events (true positive bins). These sites commonly feature low vegetation density, bare or sparsely covered soil, and low EC‐measurement heights, characteristics typical of (xeric) shrublands, savannas, but seasonally also temperate agricultural regions. Our findings are consistent with past site‐level observations of SVA, though few studies have drawn general conclusions about vegetation structure. For instance, SVA has been reported in Namib hummocks (Kool et al. 2021), Tanzanian and Greek rangelands (Kaseke et al. 2012; Kosmas et al. 2001), and Mediterranean agro‐ecosystems such as *Dehesas*, olive groves, and vineyards (Paulus et al. 2022; Verhoef et al. 2006; Kosmas et al. 2001). These landscapes often share structural features, including evergreen or perennial woody vegetation interspersed with bare or intermittently vegetated soil, typical of shrublands and savannas. SVA has also been reported for agricultural and forested sites (Paulus et al. 2024; Zhang et al. 2019; Qubaja et al. 2020), supporting our findings of variability within different vegetation structures.

While our analysis highlights clear patterns of SVA across various vegetation structures, it is crucial to recognise that ecosystems that are most likely to exhibit frequent SVA, particularly hyper‐arid and arid environments, are underrepresented in our dataset. SVA has been observed in such systems, including sandy deserts and dunes (Zhuang and Zhao 2017; Saaltink et al. 2020; Louge et al. 2022), as well as biocrust‐covered surfaces (Lopez‐Canfin et al. 2022a; Uclés et al. 2013; Li et al. 2021). However, FLUXNET includes few sites in truly hyper‐arid or desert environments. Most EC towers in our study are located in regions with higher aridity indices (AI > 0.2), and even among the arid sites included, few are situated in sparsely vegetated or desert‐like landscapes (Figure S1).

This spatial bias presents a structural limitation when it comes to assessing SVA on a global scale. Several factors compound this issue: meteorological conditions at many EC sites do not favor SVA; night‐time flux measurements may be too short or uncertain for detection; and the vertical placement of EC sensors, which are often located above dense canopies, may limit the sensitivity to near‐surface vapor fluxes, as demonstrated at the Majadas de Tiétar site.

Nevertheless, drylands cover approximately one‐third of the Earth's terrestrial surface (Maestre et al. 2021), and temporarily bare soils account for 32% of the global land area (Demattê et al. 2020). These figures suggest that the global potential for SVA is substantial, and that current flux tower networks provide only a narrow window into this phenomenon.

### 5.2 Rationale for Global Monitoring of Soil Water Vapor Adsorption

SVA, as a mechanism in drylands, remains largely unaccounted for in both observational networks and models. As a subtle, sub‐daily source of moisture, SVA plays a potentially overlooked role in triggering biogeochemical reactions under water‐limited conditions. Emerging evidence suggests that SVA may influence not only the water and carbon cycles, but also dust emissions and the potential for ecological restoration in degraded drylands.

At the land–atmosphere interface, SVA likely contributes to the persistent underestimation of bare‐soil evaporation in column‐scale soil and land surface models (Assouline et al. 2013; Balugani et al. 2021; Katata et al. 2007; Garcia Gonzalez et al. 2012). Water vapor adsorbed during the night forms thin liquid films around soil particles. These films will partly or mostly evaporate during the following day, although some bound water is likely to remain due to strong adsorptive forces—particularly in soils with high clay content or clay minerals with high specific surface area, such as smectite. Field observations show that daytime evaporation is strongly correlated with the volume of adsorbed water (Kool et al. 2021; Agam and Berliner 2004; Qubaja et al. 2020; Uclés et al. 2016). In drylands, where moisture pulses are key drivers of microbial and plant activity, nocturnal humidity pulses have been shown to stimulate ${\mathrm{CO}}_{2}$ release (Grünzweig et al. 2022; Lopez et al. 2018). SVA likely plays a key role in this process by promoting litter decomposition and microbial activation at night (Dirks et al. 2010; Evans et al. 2019; McHugh et al. 2015). Notably, in contrast to the typical temperature‐driven soil respiration patterns (Yvon‐Durocher et al. 2012; Johnston et al. 2021), peak soil ${\mathrm{CO}}_{2}$ emissions in drylands often coincide with periods of high *RHa* and low surface temperatures (Wang et al. 2014; Song et al. 2015). This counterintuitive pattern has been explained by McHugh et al. (2015), who show that under high *RHa,* atmospheric water vapor adsorbs into the soil. The resulting increase in near‐surface moisture stimulated microbial respiration. In other cases, SVA can even support nocturnal ${\mathrm{CO}}_{2}$ uptake through thin water films that facilitate dissolution and degassing of ${\mathrm{CO}}_{2}$ under diel temperature and pH fluctuations (Lopez‐Canfin et al. 2022b). This mechanism may represent a non‐negligible flux in the global carbon budget (Kim et al. 2024).

Beyond biogeochemistry, SVA may contribute to dust suppression by enhancing interparticle cohesion (Dupont 2022), and to soil restoration via hydration and stabilization of surface layers (Kim and Or 2017). Biocrusts, mosses, and lichens common in arid ecosystems can amplify the amount of water adsorbed (Li et al. 2021), improving both soil stability and microhabitat conditions—critical elements in ecosystem regeneration.

Importantly, SVA is not restricted to drylands. Observations from central Germany show that SVA can occur even in temperate ecosystems during droughts and periods of exposed bare soil (Paulus et al. 2024). This indicates that SVA can arise whenever near‐surface vapor pressure gradients reverse—during diurnal cycles, dry spells, or hot‐drought events. As the climate warms, spatial and temporal patterns of SVA are likely to change. Projected declines in soil moisture (Zhou et al. 2021), altered near‐surface humidity dynamics (Novick et al. 2024), increasing bare‐soil fractions (Grünzweig et al. 2022), and more frequent and prolonged droughts (Allen et al. 2018; Feldman et al. 2024) suggest that SVA may become more widespread. To capture these shifts, long‐term and spatially resolved monitoring is essential.

### 5.3 Eddy Covariance Method as a New Scale of Observations of Soil Water Vapor Adsorption

The EC method fundamentally differs from traditional point‐scale approaches used to measure SVA, such as lysimeters, which typically contain only bare soil (Kidron and Starinsky 2019; Kool et al. 2021; Uclés et al. 2013). EC measurements are taken above the canopy, capturing the net water vapor exchange of the entire ecosystem. This shifts the perspective from localized soil measurements to an integrated ecosystem‐scale signal, which includes transpiration, evaporation, condensation, and adsorption.

In addition to soil, various plant components—such as leaves (Malik et al. 2014; Price and Clark 2014; Burkhardt and Hunsche 2013; Dawson and Goldsmith 2018; Berry et al. 2019), bark (Lintunen et al. 2021; Gimeno et al. 2022; Mason Earles et al. 2016; Oberhuber et al. 2020), litter (Evans et al. 2019; Dirks et al. 2010; McHugh et al. 2015), and deadwood (even in temperate ecosystems, see Floriancic et al. 2023) are hygroscopic and can adsorb atmospheric moisture. This adsorption to other hygroscopic or porous materials also contributes to the EC signal, although likely to a lesser extent than the soil. The micro‐environment around the leaf surfaces tends to maintain high relative humidity due to transpiration, even during the day, which buffers leaf hygroscopic wetness against diurnal changes in *RHa* (Burkhardt and Hunsche 2013). Furthermore, the total surface area of plant tissues is much smaller than that of soil, which can reach up to 450 m^2^ per gram (Yan et al. 2023). Thus, soil likely provides a far greater surface area for vapor adsorption and rehydration during the night compared to vegetation tissues.

At the representative elementary volume scale, SVA is primarily influenced by soil properties such as clay content, clay mineralogy, and organic matter (Orchiston 1954; Hillel 2012; Arthur et al. 2014b, 2015). Clay content, in particular, enhances adsorption even at the scale of a few kilometers (Kosmas et al. 1998; Uclés et al. 2015). Yet across broader climatic gradients, climatological variables appear to be stronger determinants of SVA occurrence than soil texture and clay mineralogy alone (Paulus et al. 2024). The discrepancy illustrates a broader point that has been noted in other ecosystem‐scale research: while mechanistic understanding at the pore or sample scale is crucial, its direct transfer to ecosystem‐level observations is not always straightforward. Many factors (e.g., vegetation dynamics, surface roughness, tower height, microclimate heterogeneity) can obscure or confound signals from soil texture and mineralogy alone. Similar scale‐transfer limitations have been documented for other biogeophysical processes, such as leaf‐to‐canopy scaling in photosynthesis or soil‐to‐ecosystem scaling in respiration. Our findings are consistent with this view, although further validation is needed. Currently available global soil texture and clay mineralogy products, such as SoilGrids, offer only coarse spatial resolution and often miss critical sub‐grid variability (Weber et al. 2024; Wankmüller and Carminati 2024). Unfortunately, for the dryland sites in FLUXNET, we found insufficient site‐level soil data to evaluate the role of clay content and mineralogy in SVA occurrence—particularly in the databases compiled by Wankmüller et al. (2024) and Shi et al. (2025). Therefore, expanding site‐specific measurements of soil texture, hydraulic properties, and clay mineralogy is essential to accurately assess their influence on the occurrence and detection of SVA across ecosystems.

In addition, under certain dryland conditions, nocturnal transpiration—though not typical—can exceed SVA by orders of magnitude (Novick et al. 2009; Ogle et al. 2012; de Dios et al. 2015; Padrón et al. 2020). In such cases, plants may access water from deeper soil layers, while SVA remains confined to the dry surface and remains undetected by the EC method, particularly at sites where it is already weak or intermittent.

Despite these limitations, a key advantage of the EC method is its ability to deliver continuous, minimally intrusive measurements at the ecosystem scale. In contrast, traditional methods—such as lysimeters (Kidron and Kronenfeld 2017), gas‐exchange chambers (Bekin and Agam 2023), or synthetic sorptive materials (Kidron et al. 2023) may introduce artifacts due to differences in heat capacity, surface properties, or sorption behavior. EC thus represents a valuable tool to upscale our understanding of SVA dynamics from point‐scale observations to whole‐ecosystem processes under real‐world conditions.

### 5.4 Evidence of Hydraulic Gradients Influencing the Direction of Ecosystem Water Vapor Fluxes

A central highlight of our study is that hydraulic gradients arising from the representative elementary volume‐scale thermodynamic equilibrium between $\Psi_{s}$ and *RHs*, as described by the Kelvin equation Equation (2), can be detected empirically in the relationship between *VWC*, near‐surface *RHa*, and the dominant *λE* flux direction in many dryland ecosystems. We show that the shape of this relationship can be inferred at the ecosystem scale without requiring direct measurements of $\Psi_{s}$ or assumptions about site‐specific retention curves.

Thereby, we show that the consequences of the Kelvin equation extend beyond the soil surface, with detectable signals several meters above the ground. This scale is particularly important because it aligns with the operating scale of many land surface and Earth system models, underscoring the relevance of the Kelvin equation for describing soil–atmosphere interactions, especially in sparsely vegetated drylands. Our findings, therefore, provide scale‐appropriate observations that can be used to evaluate and improve model representations of soil–atmosphere exchange processes.

At the same time, our analysis reveals that ecosystem‐scale controls differ from those identified at the representative elementary volume scale. For the effect of soil texture and mineralogy, we confirmed that while they are key controls on adsorption processes at the representative elementary volume scale, they alone could not explain whether the process can be detected at the ecosystem level; additional factors such as aridity or PFT were also necessary. This finding suggests that factors related to vapor transport efficiency may override textural controls at larger scales.

Finally, we emphasize that uncertainties remain. In particular, it is still unclear whether SVA is absent under certain environmental conditions, or merely undetectable by EC. This ambiguity is illustrated by the contrasting results observed at different tower heights at the Majadas de Tiétar site, highlighting the need for more vertically resolved flux measurements.

### 5.5 Theoretical and Methodological Uncertainty and Limitations

As $\Psi_{s}$ and *RHs* are not part of the standard measurement suite at EC sites, we used two proxy variables for assessing the direction of the vapor gradient that are routinely available: *VWC* and *RHa*. While $\Psi_{s}$ and *RHs* are physically linked through the Kelvin equation Equation (2), no such direct, bidirectional relationship exists between *VWC* and *RHa*. *VWC* in the field reflects complex interactions among precipitation, soil texture, root water uptake, atmospheric demand, and water (vapor) transport. *RHa* is influenced by temperature, surface evaporation, and air mass dynamics.

Both proxies are also subject to sensor‐related uncertainties. For *RHa*, these include potential saturation effects in thermo‐hygrometers at high humidity. For *VWC*, FDR and TDR probes may be affected by temperature and salinity, lack of site‐specific calibration, and differential sensitivity to free versus adsorbed water (Wraith and Or 1999; Skierucha 2011). The sensor installation itself introduces additional uncertainty, as the sampling depth and location may not be representative of the ecosystem as a whole.

Although these are significant limitations that must be considered, we believe the analysis remains robust. Our conclusions are based on the consistency and relative behavior of the observed patterns across sites rather than on the absolute values of *VWC* and *RHa*. In particular, the fluctuations and transitions captured by *VWC* sensors—even at low values (< 10%)—clearly reflect changing vapor flux direction. These patterns align with theoretical expectations that SVA processes are confined to extremely dry soil conditions.

In addition, EC *λE* flux measurements have random uncertainties that depend on a combination of sampling error due to natural variability in the turbulence and sensor noise (Langford et al. 2015). A key challenge in measuring fluxes with EC at night is that the magnitude of the flux is often only slightly higher than the random error of the instrument (Paulus et al. 2024; Hollinger and Richardson 2005; Padrón et al. 2020). Nights are typically characterized by stable atmospheric conditions and intermittent, unsteady turbulence (Wohlfahrt et al. 2005), resulting in an extremely low signal‐to‐noise ratio. Although *λE* fluxes are known to be underestimated under these conditions, the impact of random noise on detecting the directionality of *λE* fluxes remains uncertain. However, since the error is random, we assume its overall effect is small. Nevertheless, a characterization of the statistical properties of the random error under nighttime conditions to make a generalized statement across ecosystems and conditions, similar to the work of Lasslop et al. (2008) or Hollinger and Richardson (2005), has not yet been performed. But the simultaneous occurrence of negative *λE* fluxes alongside independent SVA observations has been validated by multiple independent methods, including microlysimeters and scintillometers (Florentin and Agam 2017), soil chambers (Qubaja et al. 2020), and large weighing lysimeters, as well as moisture profiles (Paulus et al. 2024). These findings support the assumptions made in our analysis.

In a previous study at the Majadas de Tiétar site (Paulus et al. 2024), we tested different u* thresholds and found that the agreement between EC and lysimeter‐based SVA detection changed only marginally. However, even before applying strict filtering, the EC recall rate was relatively low (53%), and additional filtering removed between 26% and 62% of half‐hourly data. Given the short data records (often only ∼3 years) at many dryland sites, further filtering would significantly reduce statistical power without guaranteeing improved reliability in flux direction classification. At present, we lack a robust framework to quantify the uncertainty of the directionality of the EC *λE* flux across the site network, largely due to limited ancillary data and the lack of reference methods.

While SVA at the pore level occurs extremely rapidly—reaching equilibrium within microseconds to at most half a minute depending on pore size and ambient saturation (Shahraeeni and Or 2012; Kim et al. 2024)—equilibration times increase substantially for larger soil samples (Arthur et al. 2014a; Schelle et al. 2013). Field studies show that SVA decreases with increasing vegetation cover (Verhoef et al. 2006; Uclés et al. 2016; Kool et al. 2021), with mulching (Kosmas et al. 1998) and stone cover (Zhang, Wang, et al. 2024). These observations suggest an important role for aerodynamic resistance, which tends to increase with denser vegetation or surface cover, is highest near the soil surface, and decreases with canopy height. This effect likely explains the influence of vegetation structure (PFT) observed in our study.

The uncertainty of thresholds defined in this study, such as the minimum number of negative *λE* flux observations required in each bin, or the fraction of negative *λE* fluxes in each bin, also needs to be assessed. Since we currently lack the means to validate the best set of parameters across sites, the exact number of sites within FLUXNET where the pattern appears cannot be definitively stated.

Atmospheric moisture originating from the ocean can advect inland, where it condenses, and plays an important role in dew and fog formation in many coastal areas (Beysens et al. 2005; Hildebrandt and Eltahir 2006; Dawson and Goldsmith 2018; Mason Earles et al. 2016; Kidron and Lázaro 2020; Weathers et al. 2020; Schween et al. 2020; Muselli and Beysens 2021). For SVA, the role of advective moisture transport remains unresolved, although it is often associated with proximity to oceans (Agam and Berliner 2006; Kool et al. 2021). This is consistent with our finding that SVA occurrence frequency is notably high at several coastal sites.

Such spatial patterns suggest that lateral moisture transport could meaningfully contribute to observed SVA signals. However, this poses a challenge for detection using EC. While short‐range advection due to local surface moisture contrasts is well documented, the advection component from mesoscale circulation can also induce persistent horizontal vapor fluxes that are not captured by point‐scale EC measurements (Stoy et al. 2013; De Roo and Mauder 2018; Mauder et al. 2024). This mechanism may be particularly relevant for coastal or semi‐arid sites (e.g., ES‐Amo, ES‐Agu, FR‐Bil), where strong temperature and humidity gradients between ocean and land exist. Therefore, lateral transport could not only contribute to SVA itself but also explain part of the underestimation of SVA frequency and magnitude by the EC method, as previously noted by Florentin and Agam (2017) and Paulus et al. (2024).

### 5.6 Considerations and Recommendations for Using Eddy Covariance for Soil Water Vapor Adsorption Detections

Our analysis demonstrates that the direction of water vapor fluxes measured by EC is not random but follows systematic patterns. With sufficient data, a clear relationship emerges between *VWC*, *RHa*, and *λE* flux direction—especially in dry regions with sparse ground cover. We attribute this pattern primarily to vapor gradients induced by soil hydraulic properties. Therefore, negative *λE* fluxes should not be automatically discarded in EC analyses, particularly under dry soil moisture conditions.

While our classification framework has highlighted the interpretability of negative *λE* fluxes, strengthening these findings and reducing uncertainties in the direction of water vapor exchange requires complementary evidence. Such evidence could come from (i) deeper analyses of existing datasets, (ii) targeted measurement campaigns with enhanced instrumentation, or (iii) process‐oriented modeling studies of vapor exchange. Reducing uncertainty can be supported using existing datasets that indicate soil–atmosphere moisture gradients, even if they do not directly measure SVA. For instance, near‐surface moisture profiles, as used by Paulus et al. (2024), provide valuable benchmarks by showing that absolute vapor concentrations were lowest at the soil surface during downward *λE* flux events. Likewise, $H_{2}O$ concentration measurements from open‐bottom chambers could also be valuable, as they allow tracking of moisture decreases after chamber closure—similar to approaches used for other greenhouse gases (Qubaja et al. 2020; Bekin and Agam 2023). A cost‐effective technology to monitor SVA conditions in situ are *RH* and temperature loggers (∼100 €), which can also be buried in the topsoil to measure *RHs* (Kool et al. 2021; Lopez‐Canfin et al. 2022a). These devices enable tracking the direction of moisture gradients. Alternatively, in situ full‐range soil water potential measurements can be used, converting $\psi_{s}$ to *RHs* using the Kelvin equation Equation (2).

Quantifying SVA currently relies primarily on lysimeter measurements (Kidron and Starinsky 2019), which must be carefully checked for heat exchange effects before data analysis (Kidron and Kronenfeld 2017; Riedl et al. 2022). Interpreting daily time series from TDR and FDR probes is problematic due to their complex temperature‐dependent electrical conductivity response, which interacts with the specific surface area and differs between free and adsorbed water (Wraith and Or 1999; Skierucha 2011).

Modeling SVA magnitudes requires multi‐phase flow and heat transport models (Saaltink et al. 2020; Du et al. 2018), which require soil water characteristic curves that accurately represent dry conditions (i.e., a double porosity approach, Saaltink et al. 2020). While these models effectively capture adsorbed water vapor magnitudes, they demand extensive measurements of soil thermal and hydraulic properties. A simpler alternative is to use humidity and temperature gradients, along with atmospheric pressure and wind speed data (Lopez‐Canfin et al. 2022b), which may be more commonly available at EC sites.

Despite remaining open questions, our study provides the first comprehensive global overview of SVA using EC data. It demonstrates that, despite inherent uncertainties, EC measurements can retrospectively reveal the occurrence of SVA across large spatial and temporal scales. However, further measurements are needed in hyperarid and arid regions where SVA is most significant. Future research should aim to validate EC observations through modeling efforts and investigate temporal and spatial shifts in the occurrence of SVA in the context of climate change.

## Author Contributions

**Sinikka J. Paulus:** conceptualization, data curation, formal analysis, investigation, methodology, project administration, visualization, writing – original draft, writing – review and editing. **Mirco Migliavacca:** methodology, supervision, writing – review and editing. **Markus Reichstein:** funding acquisition, methodology, supervision. **Rene Orth:** supervision, writing – review and editing. **Sung‐Ching Lee:** data curation, funding acquisition, supervision, writing – review and editing. **Arnaud Carrara:** data curation, writing – review and editing. **Anke Hildebrandt:** conceptualization, methodology, supervision, writing – review and editing. **Jacob A. Nelson:** data curation, funding acquisition, supervision, writing – review and editing.

## Conflicts of Interest

The authors declare no conflicts of interest.

## Supporting information


**Data S1:** gcb70547‐sup‐0001‐supinfo.pdf.

## Data Availability

The EC data used in the results is openly available from one of five different sources. The FLUXNET2015 dataset is available from the FLUXNET Data Portal: https://fluxnet.org/data/fluxnet2015‐dataset/. ICOS data are available from the ICOS Carbon Portal, including ICOS Level 2 data: https://data.icos‐cp.eu/portal/. The ICOS Drought‐2018 dataset is available at: https://doi.org/10.18160/YVR0‐4898, and the ICOS Warm Winter 2020 dataset at: https://doi.org/10.18160/2G60‐ZHAK. Additional EC data as of December 2022 were downloaded from AmeriFlux: https://ameriflux.lbl.gov/data/aboutdata/. The soil texture data supporting this study are available from: https://files.isric.org/soilgrids/ (last access: 27 May 2025). The aridity index dataset is available in Figshare: https://doi.org/10.6084/m9.figshare.7504448.v3. Coastline data are available in Zenodo: http://doi.org/10.5281/zenodo.13943678. The FluxnetEO dataset is available from the ICOS ERIC Carbon Portal: http://doi.org/10.18160/0KWD‐3RRW. Soil hydraulic characteristics data are available from GFZ Data Services: https://doi.org/10.5880/fidgeo.2023.012. Major soil clay mineral data are available from: https://doi.pangaea.de/10.1594/PANGAEA.868929. Majadas de Tietar site‐level EC subcanopy tower data and WP4C measurements are available from the corresponding author upon reasonable request. The data that support the central findings of this study are openly available in Zenodo at https://doi.org/10.5281/zenodo.17185798.
