# Supplementary material for: Insights Into Water Vapor Uptake by Dry Soils Using a Global Eddy Covariance Observation Network
Source: Glob Chang Biol. 2025 Oct 13;31(10):e70547. doi: 10.1111/gcb.70547 (PMC12516670; doi:10.1111/gcb.70547)
Supplement: Supplementary file 1 — Data S1: gcb70547‐sup‐0001‐supinfo.pdf. [file GCB-31-e70547-s001.pdf]

Supplementary Materials for

Insights into Water Vapor Uptake by Dry Soils using a Global Eddy Covariance Observation Network

Sinikka J. Paulus[1,2,3], Mirco Migliavacca[1,4], Markus Reichstein[1], Rene Orth[3], Sung-Ching Lee[1], Arnaud Carrara[5], Anke Hildebrandt[6,2], and Jacob A. Nelson[1]

[1]Biogeochemical Integration, Max Planck Institute for Biogeochemistry, Jena, Germany
[2]Institute of Geoscience, University of Jena, Jena, Germany
[3]Faculty of Environment and Natural Resources, University of Freiburg, Germany
[4]European Commission, Joint Research Centre (JRC), Ispra, Italy
[5]Fundacion Centro de Estudios Ambientales del Mediterráneo (CEAM), Valencia, Spain
[6]Department Hydrosystemmodellierung, Helmholtz Centre for Environmental Research - UFZ, Leipzig, Germany

1 List of Symbols

Table S1: List of Symbols.

Symbol	Full form	Unit
M_w	Molecular weight of water = 0.018	$\mathrm{kg\,mol^{-1}}$
R	Universal gas constant = 8.314	$\mathrm{J\,mol^{-1}\,K^{-1}}$
T	Temperature	°C
T_a	Air temperature	°C
VWC	Volumetric Soil Water Content	$\mathrm{m\,m^{-3}}$
ρ_w	Density of water at 20°C = 998.2	$\mathrm{m\,m^{-3}C}$
RHa	Relative humidity of the near-surface atmosphere	%
RHs	Relative humidity of the soil pore space	%
RH	Relative humidity	%
VPD	Vapor Pressure Deficit	kPa
$VWC_{RH\,80}$	Texture based apparent Ecosystem Vapor Equilibrium, named based on the VWC in equilibrium with air at 80 % RH which corresponds to approximately to a water potential of - 300 000 hPa	%
Ψ_a	Water potential of the air above the soil	hPa
Ψ_w	Total soil water potential constituted of matric, chemical, and pressure potential	hPa
λE	Latent heat flux	$\mathrm{W\,m^{-2}}$
σ_{eff}	Effective transport parameter	Unitless
e_0	Water vapor pressure of free water at saturation, as defined by the WMO (2008); also known as equilibrium vapor pressure	kPa
e	Ambient water vapor pressure	kPa
u^*	Friction velocity	$\mathrm{m\,s^{-1}}$

2 Station location of eddy covariance towers

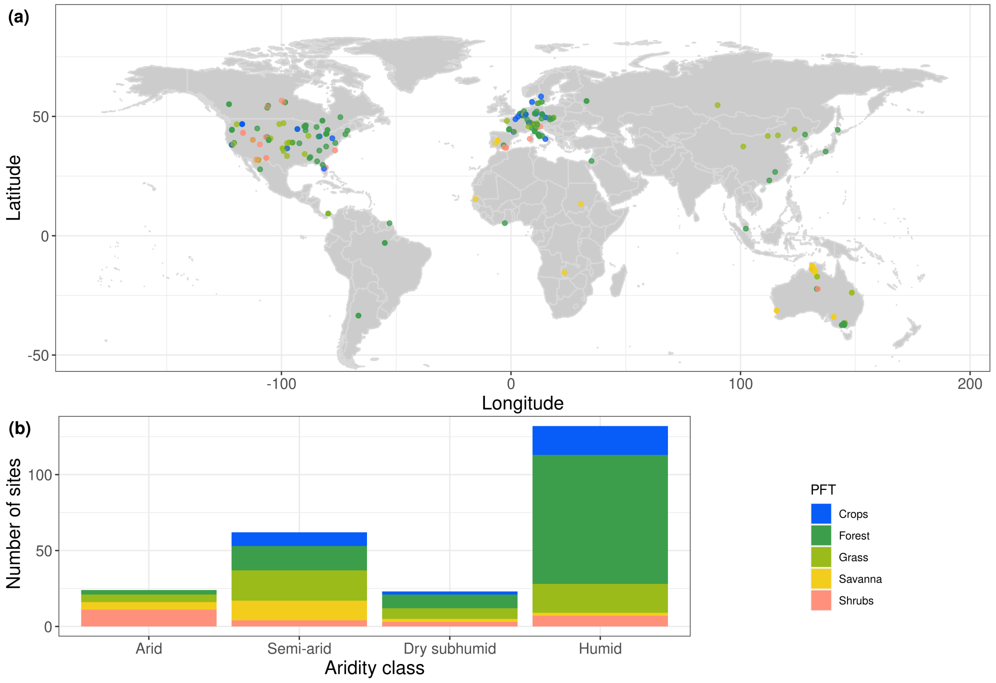

Figure S1: (a) Geographic distribution of the 229 eddy covariance sites included in this study and (b) number of sites per aridity class. The colors indicate plant functional types (PFT), classified according to the International Geosphere–Biosphere Programme (IGBP) scheme.

3 Dryland suptype classification

Table S2: Climate classification and dryland subtypes based on the Aridity Index (Thomas et al., 1997).

Aridity Index (AI) values	Climate classification
AI < 0.05	Hyper-arid
0.05 < AI < 0.2	Arid
0.2 < AI < 0.5	Semi-arid
0.5 < AI < 0.65	Dry sub-humid

4 Effect of temperature on relative humidity in the soil

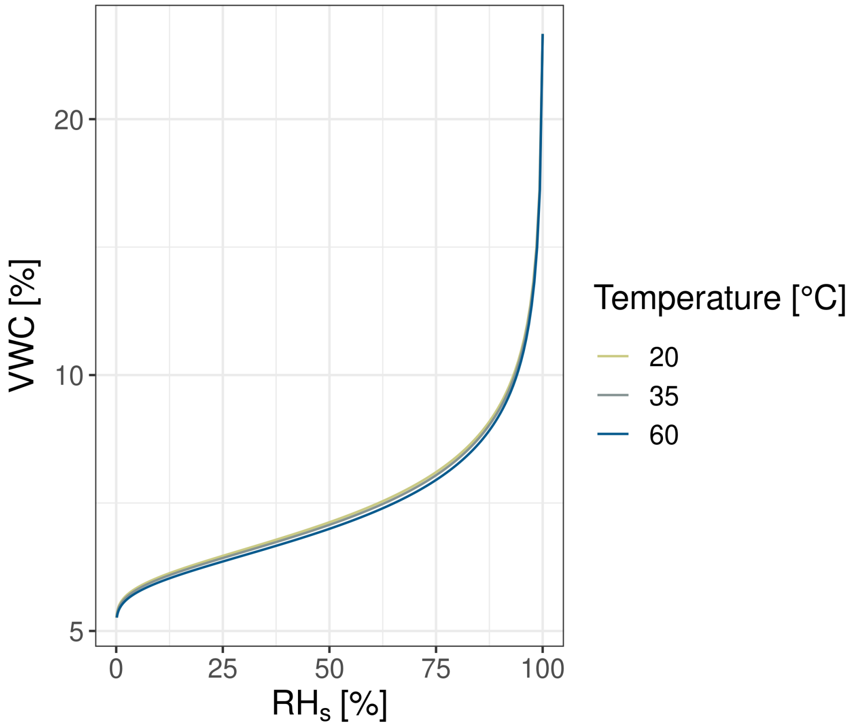

Figure S2: Influence of temperature on the relationships between volumetric soil water content (VWC) and relative humidity in the soil pores (RHs), converted from soil water potential using the Kelvin equation (Equation 3.2) for 20, 35, and 60 $^{\circ}C$.

5 Soil water retention data

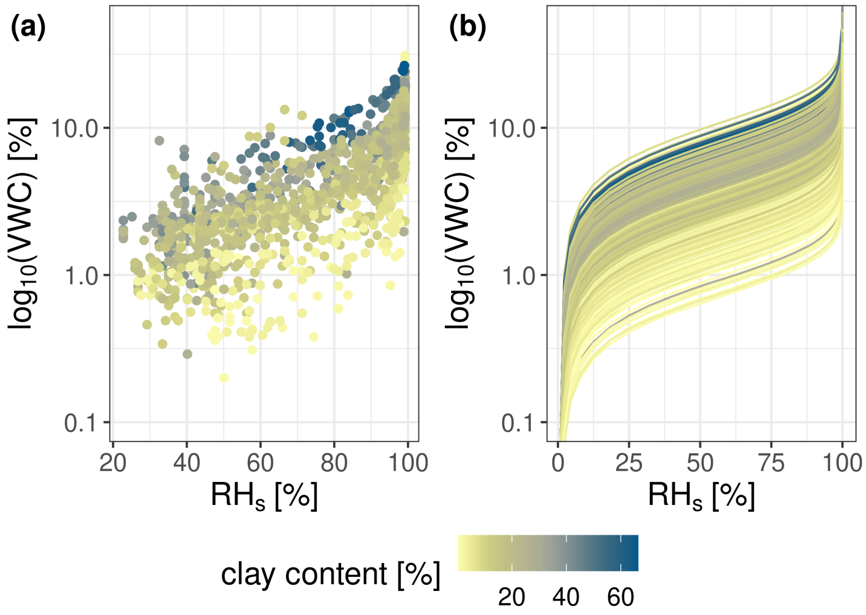

Figure S3: Relationships between the logarithm (base 10) of volumetric soil water content ($\log_{10}(VWC)$) and relative humidity in the soil pores (RHs), converted from soil water potential using the Kelvin equation. The data are from 542 soil samples from Hohenbrink et al. (2023). Panel (a) represent measured values, and panel (b) shows the interpolated relationships for the respective samples based on the Peter-Iden-Durner Model. The colorcode illustrates the clay fraction of each soil sample.

6 Apparent Ecosystem Vapor Equilibrium relationship with texture

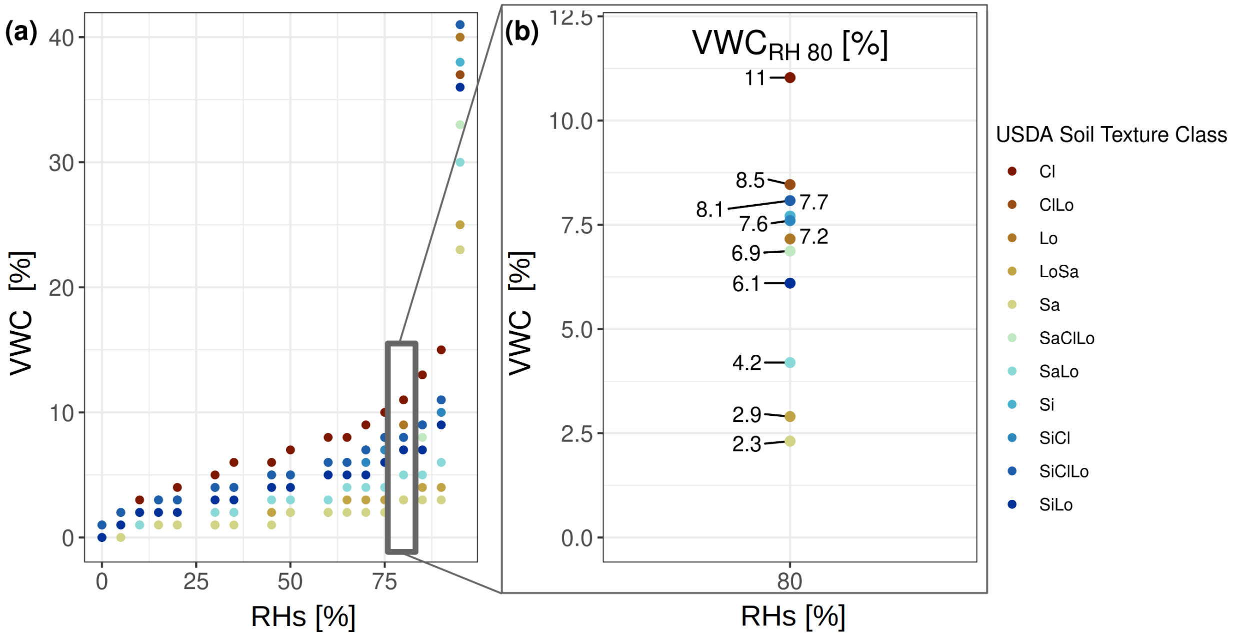

Figure S4: Average relationships between volumetric soil water content (VWC) and relative humidity in the soil pores (RH_s), converted from soil water potential using the Kelvin equation for different soil textures. Panel (a) illustrates the full range of RH_s while panel (b) zooms in to the range of VWC values at 80 % RH only.

7 Definition of metrics for classification evaluation

Accuracy: Binary accuracy (ACC) is the proportion of correct classifications (both true positives and true negatives) relative to all classifications.

$$\text{ACC} = \frac{TP + TN}{TP + TN + FP + FN}$$

True Positive Fraction: True Positive Fraction (TPF, also called Sensitivity or Recall) is the proportion of true positives among the total actual positives.

$$\text{TPF} = \frac{TP}{TP + FN}$$

True Negative Fraction: True Negative Fraction (TNF, also called Specificity) is the proportion of true negatives among the total actual negatives.

$$\text{TNF} = \frac{TN}{TN + FP}$$

False Positive Fraction: The False Positive Fraction (FPF) is the proportion of false positives among the total actual negatives.

$$\text{FPR} = \frac{FP}{FP + TN}$$

8 Distributions of classification metrics across EVEa

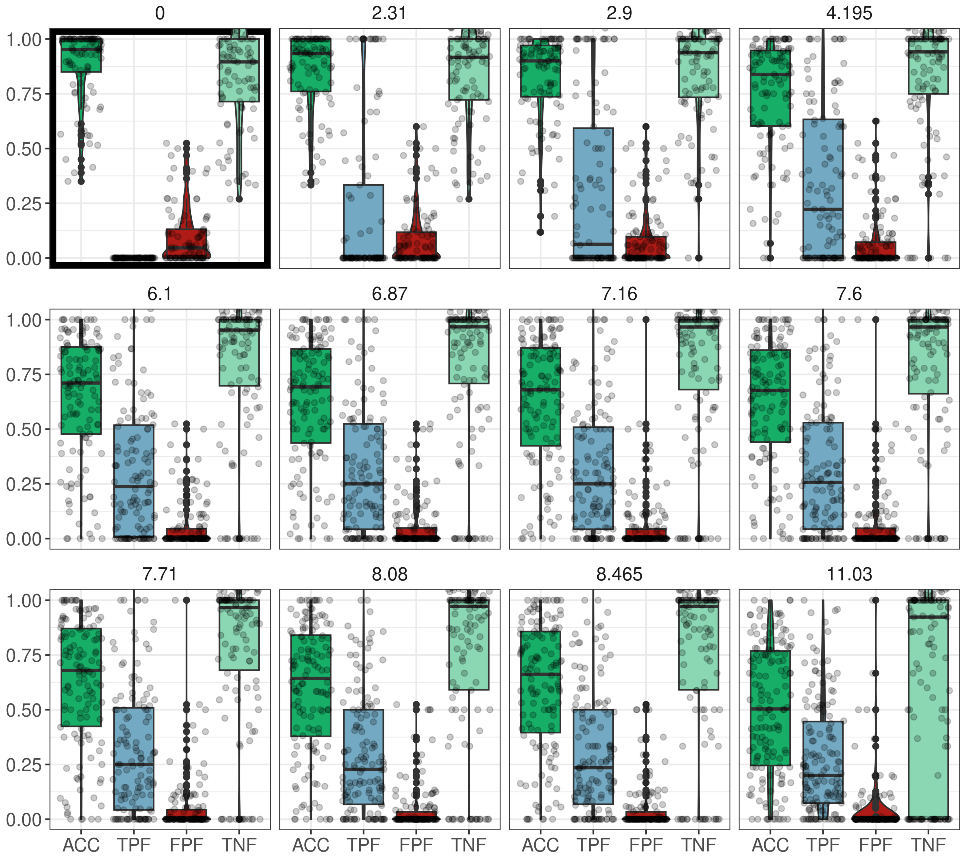

Figure S5: Classification metrics across sites for each apparent Ecosystem Vapor Equilibrium (EVEa) template: Accuracy (ACC), True Positive Fraction (TPF), False Positive Fraction (FPF), and True Negative Fraction (TNF). Each panel corresponds to one EVEa template, defined by volumetric soil water content at 80% relative humidity under laboratory equilibrium conditions (e.g., 2.3 for sand, 11.03 for clay, based on USDA texture classification). The value 0 represents the assumption that no systematic negative latent heat (λE) fluxes are detectable in EC data—i.e., no texture influence.

9 EC sites with high accuracy improvements

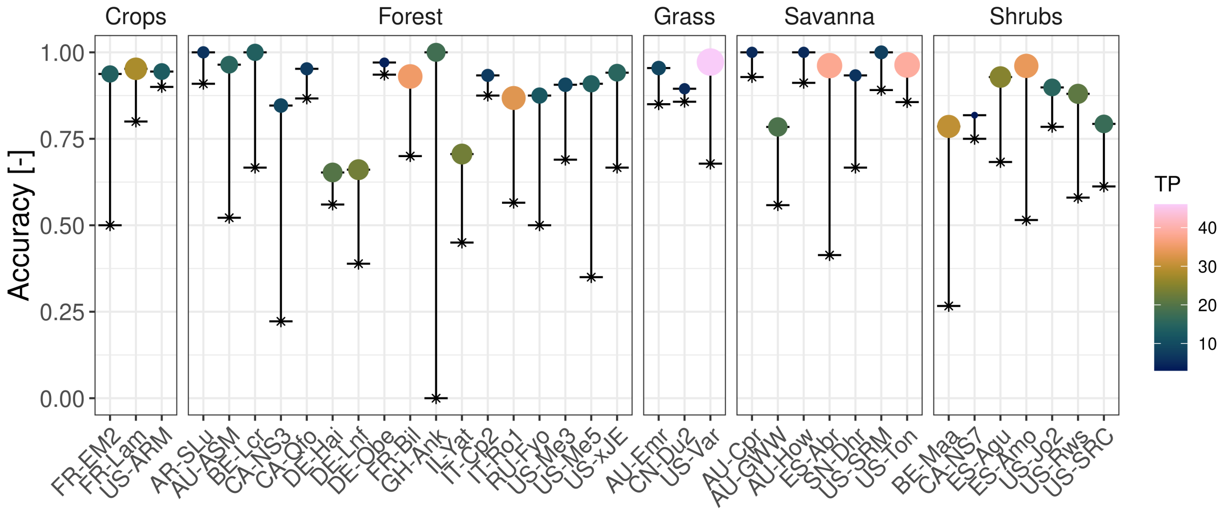

Figure S6: Illustration of sites with an accuracy improvement of more than 3 % compared to the null hypothesis (no dominant flux direction, marked in the figure by asterisks). Colors represent true positive (TP) bins for each site, determined by the combination of local climate and EC detection.

10 Effect of sand content and clay mineralogy on accuracy

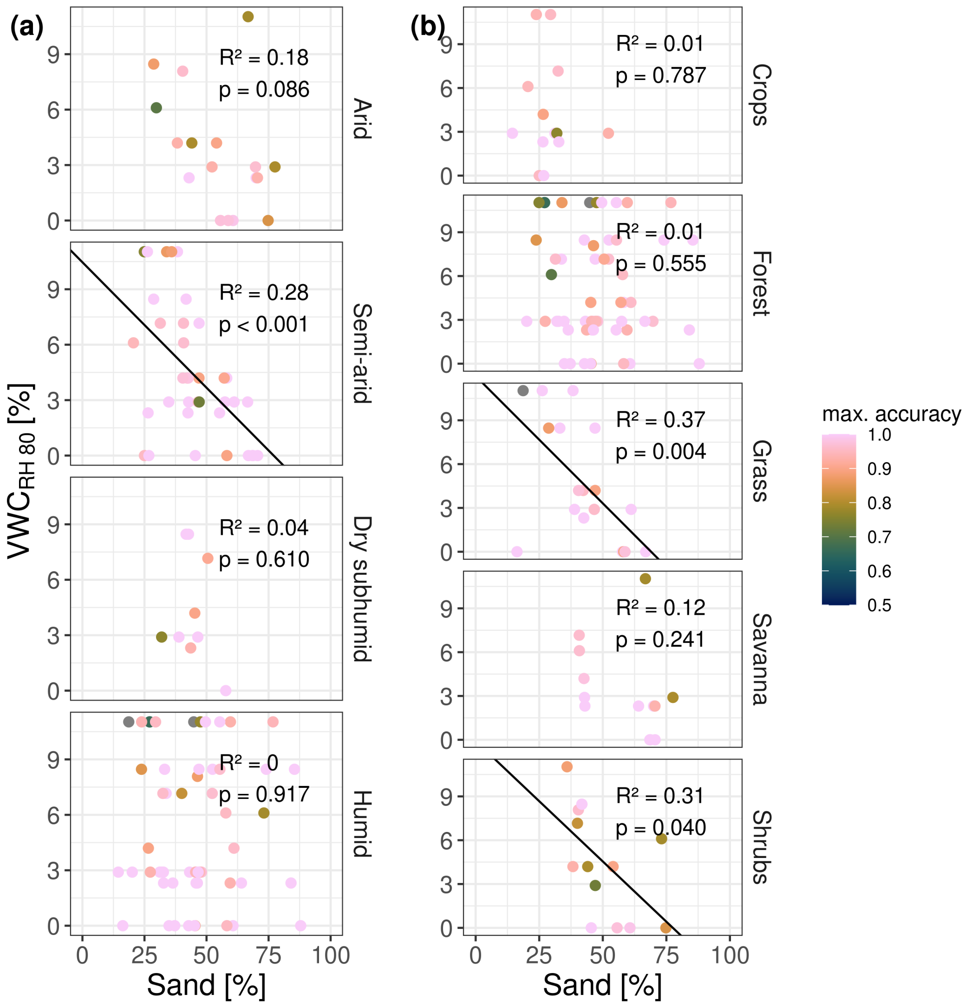

Figure S7: Relationship between the apparent Ecosystem Vapor Equilibrium (EVEa) and sand content across EC sites. EVEa is defined as the volumetric water content (VWC) at 80% relative humidity (RH) associated with the highest classification accuracy of flux direction for each EC site. Each point represents an EC site, grouped by (a) aridity class and (b) plant functional type. Black lines indicate significant linear relationships (p¡0.05); R^2 and p-values are shown in each panel. Point color denotes classification accuracy.

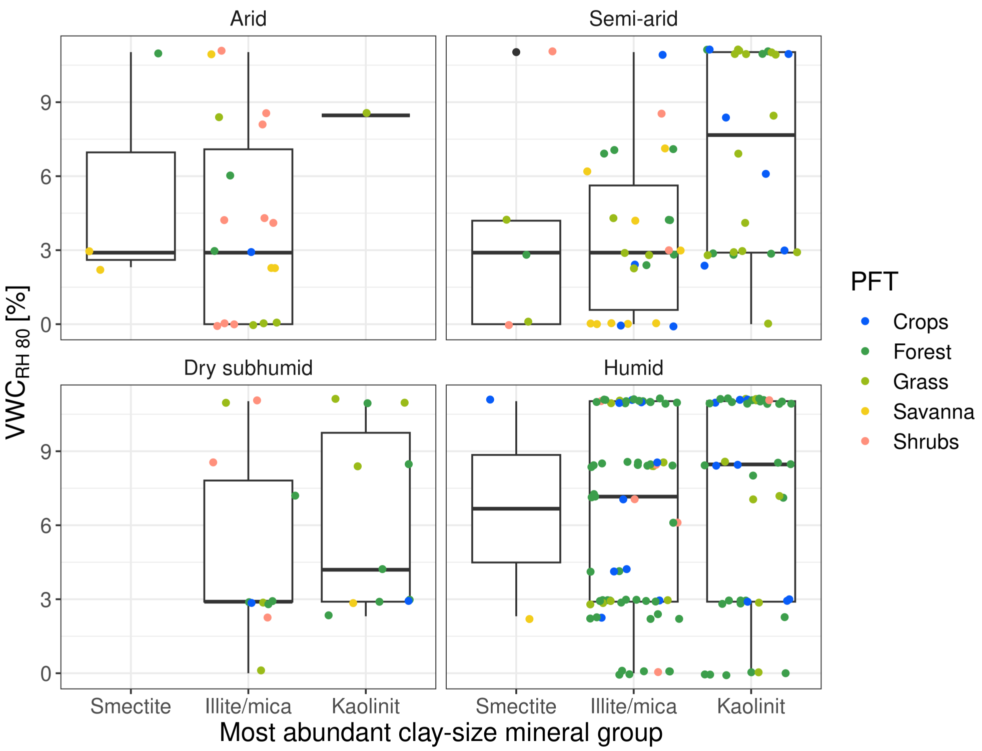

Figure S8: Relationship between the apparent Ecosystem Vapor Equilibrium (EVEa) and most-abundant clay-size mineral group across EC sites. EVEa is defined as the volumetric water content (VWC) at 80% relative humidity (RH) associated with the highest classification accuracy of flux direction for each EC site. Each point represents an EC site. Point color denote the Plant Function Types (PFT).

11 Coastal proximity, aridity, and vegetation effects on SVA occurrence

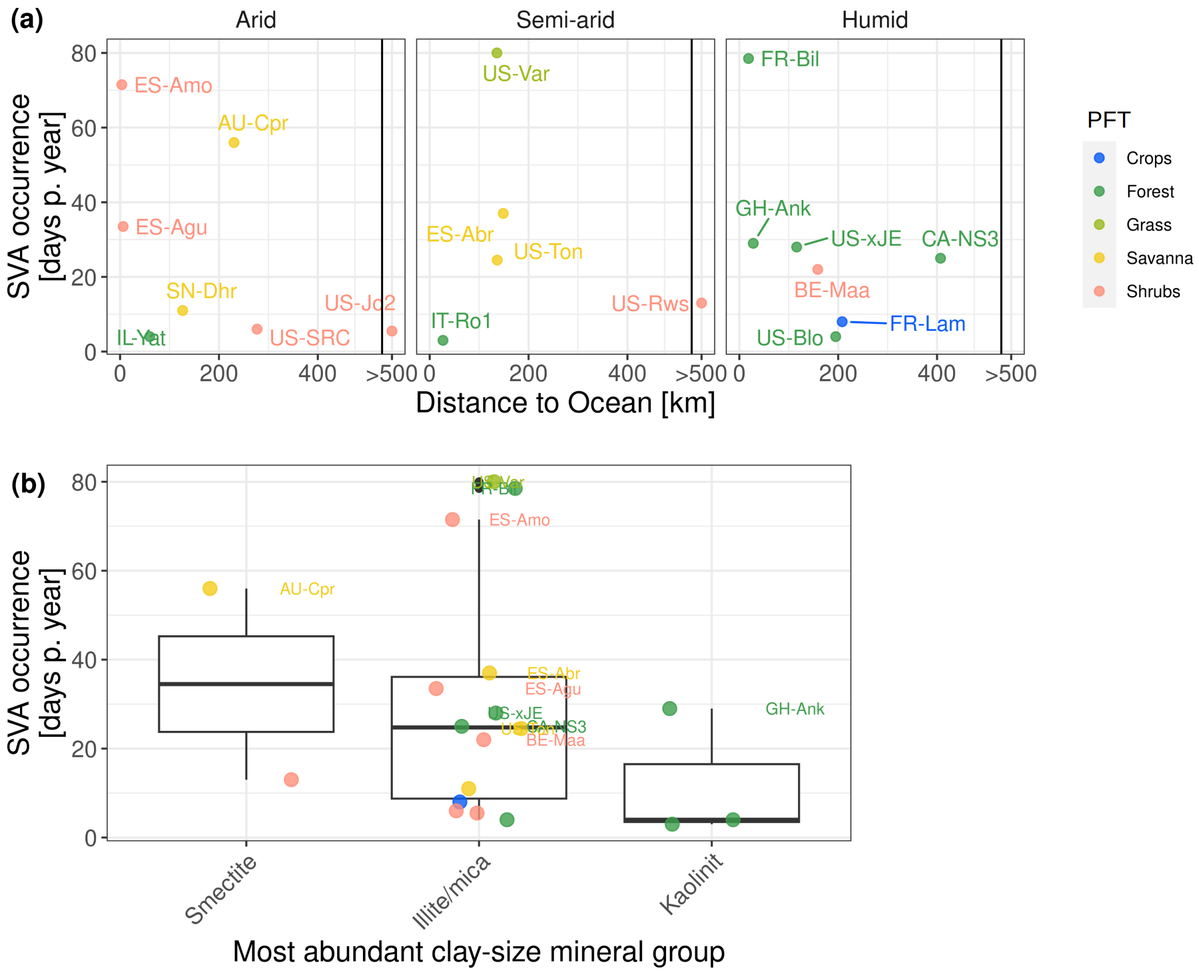

Figure S9: Effect of (a) distance from the coast (grouped at ≥500 km) across aridity classes and (b) dominant clay-size mineral group on annual soil water vapor adsorption (SVA) frequency, expressed as days per year. Point color indicates Plant Functional Types (PFTs). Only sites with at least one year exhibiting ≥10 days with ≥3 hours of SVA are included.

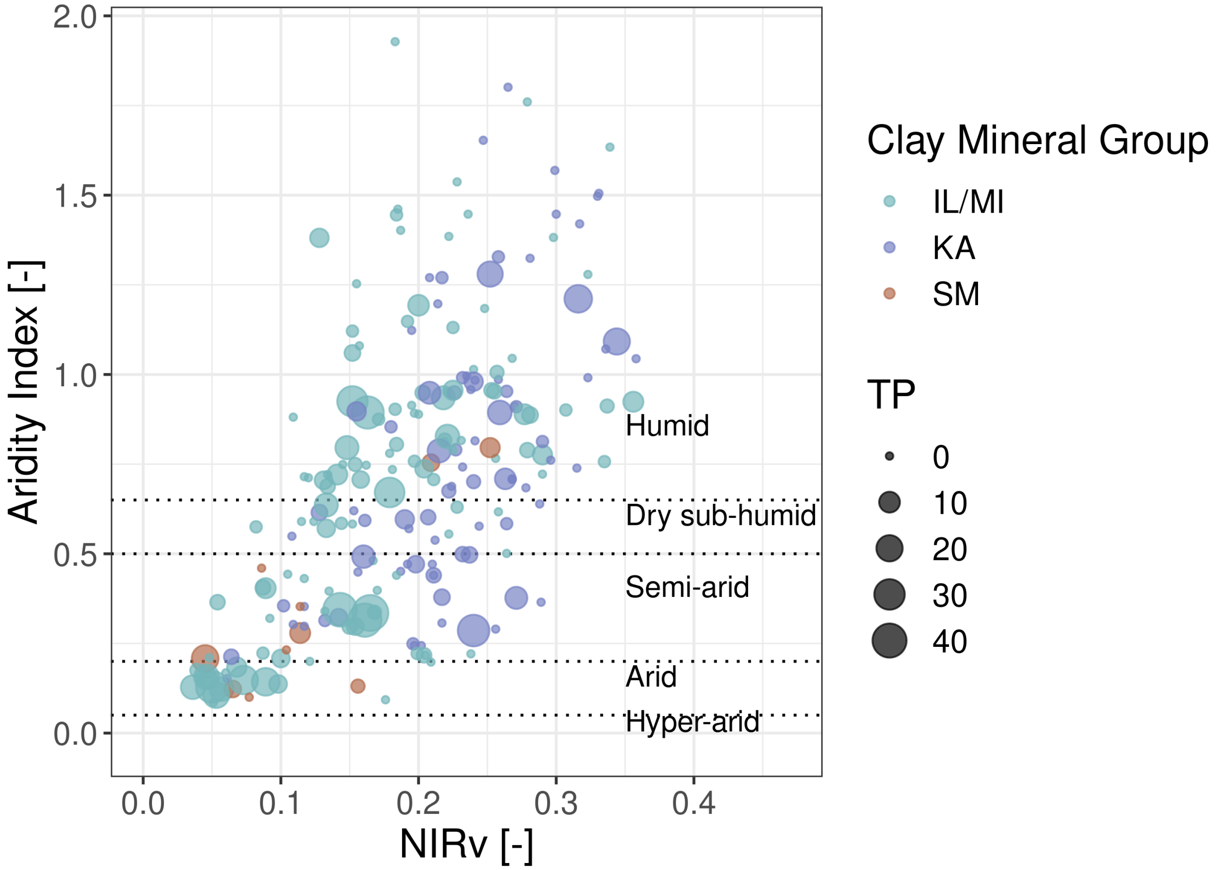

Figure S10: Relationship between NIRv and the Aridity Index across FLUXNET sites. Each point represents one site; point size reflects the number of true positive (TP) instances of SVA occurrence, and point color indicates the dominant clay-size mineral group in the topsoil. Horizontal dotted lines denote thresholds separating aridity classes.

12 Selected images of eddy covariance sites

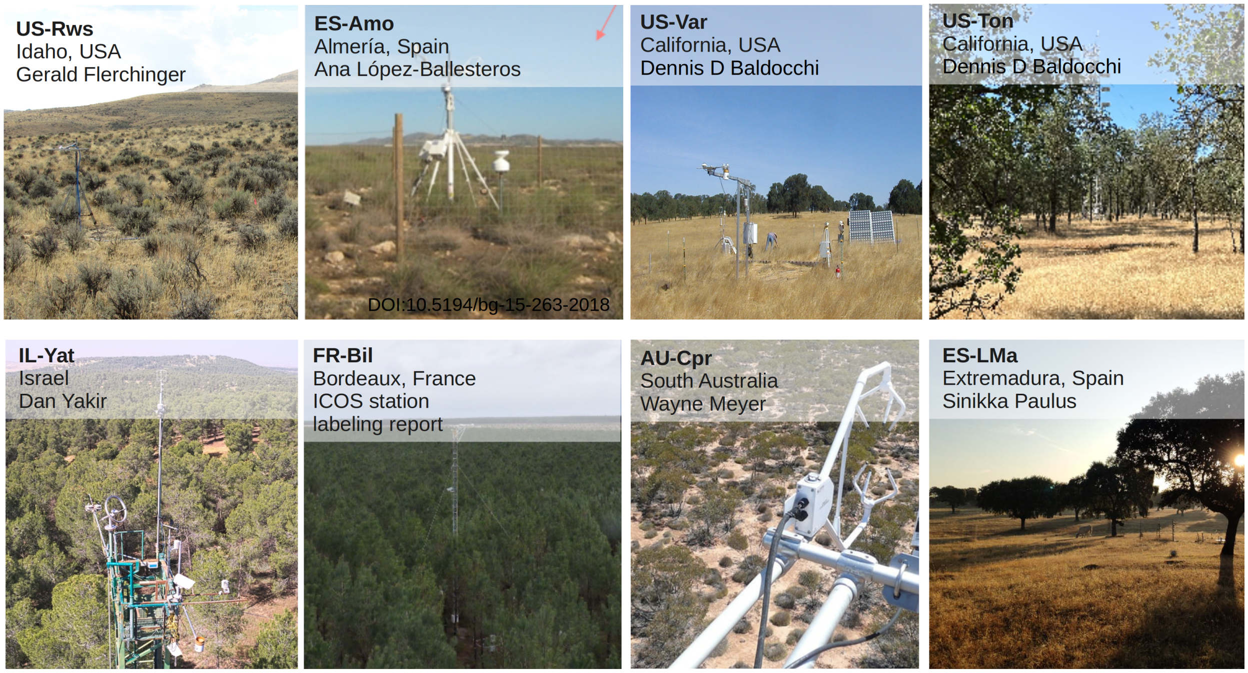

Figure S11: Selected images of eddy-covariance tower sites exhibiting a strong negative latent heat pattern under low soil moisture conditions. Each site has minimum 15 true positive instances and is among the sites with frequent soil vapor adsorption occurrence. Fluxnet station IDs, locations, and photo credits are provided in the top-left corner of each image.

13 Site list and ancillary data

Table S3: Site list and ancillary data.

Site	AI	Aridity	IGBP	NIRv	Mineral	Sand	dist. coast	TP	DpY	HpD
AR-SLu	0.208	Semi-arid	MF	0.100	IL/MI	58.1	601.6	6.0	NaN	NaN
AT-Neu	1.184	Humid	GRA	0.248	IL/MI	40.9	194.4	0.0	NaN	NaN
AU-Ade	0.620	Dry subhumid	WSA	0.153	KA	63.2	58.5	0.0	NaN	NaN
AU-ASM	0.123	Arid	ENF	0.057	IL/MI	69.7	832.1	15.0	NaN	NaN
AU-Cpr	0.123	Arid	SAV	0.065	SM	70.0	230.4	5.0	56.0	4.500
AU-DaP	0.501	Dry subhumid	GRA	0.264	IL/MI	57.8	123.5	0.0	NaN	NaN
AU-DaS	0.481	Semi-arid	SAV	0.167	IL/MI	68.7	135.6	0.0	NaN	NaN
AU-Dry	0.340	Semi-arid	SAV	0.132	IL/MI	70.6	294.3	0.0	NaN	NaN
AU-Emr	0.279	Semi-arid	GRA	0.114	SM	42.3	188.3	9.0	NaN	NaN
AU-Gin	0.320	Semi-arid	WSA	0.092	IL/MI	70.8	16.5	0.0	NaN	NaN
AU-GWW	0.106	Arid	SAV	0.053	IL/MI	66.8	472.0	19.0	NaN	NaN
AU-How	0.754	Humid	WSA	0.209	SM	64.1	18.2	5.0	NaN	NaN
AU-Lox	0.131	Arid	DBF	0.156	SM	65.0	194.1	2.0	NaN	NaN
AU-RDF	0.396	Semi-arid	WSA	0.135	IL/MI	68.5	278.5	0.0	NaN	NaN
AU-Rig	0.365	Semi-arid	GRA	0.289	KA	61.1	144.1	0.0	NaN	NaN
AU-Rob	1.569	Humid	EBF	0.299	KA	46.9	22.8	0.0	NaN	NaN
AU-Stp	0.232	Semi-arid	GRA	0.104	SM	58.2	331.3	0.0	NaN	NaN
AU-TTE	0.085	Arid	OSH	0.051	IL/MI	74.8	817.9	0.0	NaN	NaN
AU-Wac	1.123	Humid	EBF	0.195	KA	57.3	51.4	0.0	NaN	NaN
AU-Whr	0.353	Semi-arid	EBF	0.117	KA	66.6	129.9	0.0	NaN	NaN
AU-Wom	0.854	Humid	EBF	0.180	KA	62.3	78.1	1.0	NaN	NaN
BE-Bra	0.949	Humid	MF	0.203	IL/MI	84.0	16.5	3.0	NaN	NaN
BE-Dor	1.045	Humid	GRA	0.268	IL/MI	18.7	120.8	0.0	NaN	NaN
BE-Lcr	0.935	Humid	DBF	0.218	IL/MI	74.1	24.5	14.0	NaN	NaN
BE-Lon	0.958	Humid	CRO	0.238	KA	14.5	90.2	0.0	NaN	NaN
BE-Maa	0.925	Humid	CSH	0.152	IL/MI	73.1	158.7	30.0	22.0	4.750
BE-Vie	1.385	Humid	MF	0.222	IL/MI	36.4	164.5	0.0	NaN	NaN
BR-CST	0.433	Semi-arid	DNF	NaN	IL/MI	54.8	344.5	1.0	NaN	NaN
BR-Sa3	1.420	Humid	EBF	0.317	KA	41.7	626.9	0.0	NaN	NaN
CA-Cbo	0.956	Humid	DBF	0.253	IL/MI	59.5	640.5	3.0	NaN	NaN
CA-Gro	0.950	Humid	MF	0.221	IL/MI	52.4	367.5	0.0	NaN	NaN
CA-LP1	0.881	Humid	ENF	0.109	IL/MI	45.4	504.8	0.0	NaN	NaN
CA-NS1	0.712	Humid	ENF	0.120	IL/MI	28.5	413.4	0.0	NaN	NaN
CA-NS2	0.715	Humid	ENF	0.117	IL/MI	28.4	415.6	0.0	NaN	NaN
CA-NS3	0.721	Humid	ENF	0.141	IL/MI	23.8	407.1	9.0	25.0	7.000
CA-NS4	0.721	Humid	ENF	0.134	IL/MI	23.7	406.8	0.0	NaN	NaN
CA-NS5	0.714	Humid	ENF	0.153	IL/MI	24.8	415.0	NaN	NaN	NaN
CA-NS6	0.707	Humid	OSH	0.158	IL/MI	30.1	426.1	5.0	NaN	NaN
CA-NS7	0.688	Humid	OSH	0.134	IL/MI	40.0	476.1	3.0	NaN	NaN
CA-Oas	0.555	Dry subhumid	DBF	0.222	IL/MI	46.5	925.2	0.0	NaN	NaN

Continued on next page

Site	AI	Aridity	IGBP	NIRv	Mineral	Sand	dist. coast	TP	DpY	HpD
CA-Qfo	1.381	Humid	ENF	0.128	IL/MI	57.8	389.6	7.0	NaN	NaN
CA-SF1	0.615	Dry subhumid	ENF	0.128	KA	49.7	841.4	4.0	NaN	NaN
CA-SF2	0.593	Dry subhumid	ENF	0.161	KA	52.8	860.8	1.0	NaN	NaN
CA-SF3	0.583	Dry subhumid	OSH	0.152	IL/MI	56.7	880.0	0.0	NaN	NaN
CA-TP1	0.986	Humid	ENF	0.258	KA	58.3	556.0	0.0	NaN	NaN
CA-TP2	0.984	Humid	ENF	0.241	KA	59.5	560.8	0.0	NaN	NaN
CA-TP3	0.991	Humid	ENF	0.232	KA	67.4	549.3	1.0	NaN	NaN
CA-TP4	0.996	Humid	ENF	0.235	KA	68.7	550.1	0.0	NaN	NaN
CA-TPD	0.991	Humid	DBF	0.323	KA	60.7	553.7	0.0	NaN	NaN
CH-Aws	1.402	Humid	GRA	0.187	IL/MI	49.4	250.5	0.0	NaN	NaN
CH-Cha	1.328	Humid	GRA	0.258	KA	36.5	310.4	1.0	NaN	NaN
CH-Dav	1.253	Humid	ENF	0.155	IL/MI	45.8	244.3	0.0	NaN	NaN
CH-Fru	1.760	Humid	GRA	0.279	IL/MI	33.1	299.3	0.0	NaN	NaN
CH-Lae	1.447	Humid	MF	0.300	KA	32.6	340.4	0.0	NaN	NaN
CH-Oe1	1.270	Humid	GRA	0.208	KA	31.7	327.3	0.0	NaN	NaN
CH-Oe2	1.270	Humid	CRO	0.217	KA	31.2	327.3	1.0	NaN	NaN
CN-Cha	0.766	Humid	MF	0.256	IL/MI	32.9	156.2	0.0	NaN	NaN
CN-Cng	0.314	Semi-arid	GRA	0.132	KA	51.4	427.6	1.0	NaN	NaN
CN-Dan	0.308	Semi-arid	GRA	0.060	IL/MI	56.7	806.7	NaN	NaN	NaN
CN-Din	1.447	Humid	EBF	0.236	IL/MI	43.2	115.0	0.0	NaN	NaN
CN-Du2	0.323	Semi-arid	GRA	0.142	KA	47.0	389.7	5.0	NaN	NaN
CN-Du3	0.315	Semi-arid	GRA	0.167	KA	46.3	390.6	NaN	NaN	NaN
CN-HaM	0.590	Dry subhumid	GRA	0.124	IL/MI	45.5	1864.2	0.0	NaN	NaN
CN-Qia	1.197	Humid	ENF	0.214	KA	32.0	398.6	0.0	NaN	NaN
CN-Sw2	0.152	Arid	GRA	0.061	KA	47.8	586.0	0.0	NaN	NaN
CZ-BK1	1.445	Humid	ENF	0.184	IL/MI	45.6	555.3	1.0	NaN	NaN
CZ-BK2	1.461	Humid	GRA	0.185	IL/MI	46.1	554.8	0.0	NaN	NaN
CZ-KrP	0.630	Dry subhumid	CRO	0.228	IL/MI	40.4	434.8	1.0	NaN	NaN
CZ-Lnz	0.639	Dry subhumid	MF	0.288	KA	54.0	411.9	0.0	NaN	NaN
CZ-RAJ	0.747	Humid	ENF	0.162	IL/MI	44.4	468.6	0.0	NaN	NaN
CZ-Stn	0.901	Humid	DBF	0.307	IL/MI	33.7	492.5	1.0	NaN	NaN
DE-Geb	0.584	Dry subhumid	CRO	0.264	KA	31.9	297.1	1.0	NaN	NaN
DE-Gri	0.889	Humid	GRA	0.200	IL/MI	40.7	329.5	0.0	NaN	NaN
DE-Hai	1.092	Humid	DBF	0.344	KA	27.1	283.0	20.0	NaN	NaN
DE-HoH	0.722	Humid	DBF	0.290	IL/MI	34.9	194.9	0.0	NaN	NaN
DE-Hzd	0.903	Humid	DBF	0.183	IL/MI	41.6	328.2	1.0	NaN	NaN
DE-Kli	1.006	Humid	CRO	0.257	IL/MI	32.4	335.7	2.0	NaN	NaN
DE-Lkb	1.928	Humid	ENF	0.183	IL/MI	47.9	365.2	0.0	NaN	NaN
DE-Lnf	1.211	Humid	DBF	0.316	KA	27.1	255.7	23.0	NaN	NaN
DE-Obe	1.060	Humid	ENF	0.152	IL/MI	48.0	345.6	4.0	NaN	NaN
DE-RuR	1.382	Humid	GRA	0.298	IL/MI	28.9	229.9	0.0	NaN	NaN
DE-RuS	0.817	Humid	CRO	0.219	IL/MI	22.5	216.9	2.0	NaN	NaN
DE-RuW	1.655	Humid	ENF	0.162	IL/MI	39.2	235.3	NaN	NaN	NaN
DE-Seh	0.813	Humid	CRO	0.219	IL/MI	22.0	216.5	0.0	NaN	NaN

Continued on next page

Site	AI	Aridity	IGBP	NIRv	Mineral	Sand	dist. coast	TP	DpY	HpD
DE-Tha	0.892	Humid	ENF	0.197	IL/MI	42.8	327.6	0.0	NaN	NaN
DK-Eng	0.789	Humid	GRA	0.279	IL/MI	55.2	14.5	3.0	NaN	NaN
DK-Fou	1.014	Humid	CRO	0.240	IL/MI	67.8	73.4	0.0	NaN	NaN
DK-Gds	1.121	Humid	ENF	0.152	IL/MI	75.6	42.8	1.0	NaN	NaN
DK-Sor	0.776	Humid	DBF	0.290	IL/MI	61.1	23.4	8.0	NaN	NaN
DK-Vng	1.131	Humid	CRO	0.225	IL/MI	74.9	44.1	1.0	NaN	NaN
ES-Abr	0.315	Semi-arid	SAV	0.161	IL/MI	40.7	148.8	38.0	37.0	4.000
ES-Agu	0.148	Arid	OSH	0.073	IL/MI	38.3	6.2	25.0	33.5	5.000
ES-Amo	0.129	Arid	OSH	0.050	IL/MI	40.4	3.5	34.0	71.5	6.500
ES-Cnd	0.200	Semi-arid	EBF	0.121	IL/MI	31.4	135.5	0.0	NaN	NaN
ES-LgS	0.575	Dry subhumid	OSH	0.082	IL/MI	44.9	38.5	1.0	NaN	NaN
ES-LJu	0.365	Semi-arid	OSH	0.054	IL/MI	41.8	20.8	3.0	NaN	NaN
ES-LM1	0.297	Semi-arid	SAV	0.150	IL/MI	42.8	310.9	3.0	NaN	NaN
ES-LM2	0.297	Semi-arid	SAV	0.154	IL/MI	42.6	310.3	5.0	NaN	NaN
FR-Aur	0.737	Humid	CRO	0.204	IL/MI	26.6	196.8	6.0	NaN	NaN
FR-Bil	0.894	Humid	ENF	0.163	IL/MI	59.6	18.6	35.0	78.5	8.000
FR-EM2	0.787	Humid	CRO	0.215	KA	23.9	138.5	14.0	NaN	NaN
FR-Gri	0.675	Humid	CRO	0.222	KA	26.0	136.3	2.0	NaN	NaN
FR-Hes	0.912	Humid	DBF	0.337	IL/MI	27.3	354.3	2.0	NaN	NaN
FR-Lam	0.671	Humid	CRO	0.179	IL/MI	29.5	208.1	28.0	8.0	3.875
FR-LBr	0.875	Humid	ENF	0.171	IL/MI	66.9	20.5	1.0	NaN	NaN
FR-Mej	0.790	Humid	GRA	0.227	KA	38.3	44.4	1.0	NaN	NaN
FR-Tou	0.585	Dry subhumid	GRA	0.144	IL/MI	38.9	139.6	1.0	NaN	NaN
GF-Guy	1.801	Humid	EBF	0.265	KA	44.9	10.9	0.0	NaN	NaN
GH-Ank	1.280	Humid	EBF	0.252	KA	55.3	28.2	18.0	29.0	6.000
IL-Yat	0.143	Arid	ENF	0.089	IL/MI	29.8	60.0	23.0	4.0	3.750
IT-BCi	0.805	Humid	CRO	0.184	IL/MI	32.7	3.0	2.0	NaN	NaN
IT-BFt	0.617	Dry subhumid	DBF	0.258	IL/MI	41.6	140.6	0.0	NaN	NaN
IT-CA1	0.244	Semi-arid	DBF	0.197	KA	31.4	33.0	0.0	NaN	NaN
IT-CA2	0.244	Semi-arid	CRO	0.202	KA	31.1	32.7	0.0	NaN	NaN
IT-CA3	0.249	Semi-arid	DBF	0.196	KA	31.3	32.7	1.0	NaN	NaN
IT-Col	0.757	Humid	DBF	0.335	IL/MI	35.9	65.4	1.0	NaN	NaN
IT-Cp2	0.596	Dry subhumid	EBF	0.190	KA	43.6	0.9	7.0	NaN	NaN
IT-Cpz	0.602	Dry subhumid	EBF	0.207	KA	45.3	2.0	3.0	NaN	NaN
IT-Isp	1.148	Humid	DBF	0.192	IL/MI	39.6	154.3	1.0	NaN	NaN
IT-Lav	0.538	Dry subhumid	ENF	0.212	KA	42.7	94.1	0.0	NaN	NaN
IT-Lsn	0.914	Humid	OSH	0.195	IL/MI	28.6	10.7	0.0	NaN	NaN
IT-MBo	0.377	Semi-arid	GRA	0.271	KA	38.3	127.5	12.0	NaN	NaN
IT-Noe	0.571	Dry subhumid	CSH	0.133	IL/MI	41.8	0.2	6.0	NaN	NaN
IT-PT1	0.758	Humid	DBF	0.197	IL/MI	43.5	88.2	1.0	NaN	NaN
IT-Ren	0.780	Humid	ENF	0.179	IL/MI	47.2	139.0	0.0	NaN	NaN
IT-Ro1	0.286	Semi-arid	DBF	0.240	KA	33.9	26.8	33.0	3.0	3.500
IT-Ro2	0.290	Semi-arid	DBF	0.256	KA	34.8	25.1	0.0	NaN	NaN
IT-SR2	0.749	Humid	ENF	0.154	IL/MI	46.3	0.3	2.0	NaN	NaN

Continued on next page

Site	AI	Aridity	IGBP	NIRv	Mineral	Sand	dist. coast	TP	DpY	HpD
IT-SRo	0.749	Humid	ENF	0.145	IL/MI	45.2	0.6	0.0	NaN	NaN
IT-Tor	1.537	Humid	GRA	0.228	IL/MI	46.6	182.1	0.0	NaN	NaN
JP-MBF	1.634	Humid	DBF	0.339	IL/MI	41.3	52.8	0.0	NaN	NaN
JP-SMF	1.653	Humid	MF	0.247	KA	37.2	29.4	0.0	NaN	NaN
MX-Tes	0.307	Semi-arid	DBF	0.217	KA	24.9	105.5	0.0	NaN	NaN
MY-PSO	1.324	Humid	EBF	0.281	KA	42.6	70.0	0.0	NaN	NaN
NL-Loo	1.080	Humid	ENF	0.157	IL/MI	87.9	97.8	0.0	NaN	NaN
PA-SPn	1.497	Humid	DBF	0.330	KA	41.1	18.8	0.0	NaN	NaN
PA-SPs	1.505	Humid	GRA	0.331	KA	40.9	19.3	0.0	NaN	NaN
RU-Fy2	0.949	Humid	ENF	0.226	KA	46.0	412.1	2.0	NaN	NaN
RU-Fyo	0.949	Humid	ENF	0.208	KA	46.4	411.9	12.0	NaN	NaN
RU-Ha1	0.549	Dry subhumid	GRA	0.108	KA	37.6	1539.4	0.0	NaN	NaN
SD-Dem	0.100	Arid	SAV	0.077	SM	77.6	1000.4	0.0	NaN	NaN
SE-Htm	1.193	Humid	ENF	0.200	IL/MI	64.3	49.5	10.0	NaN	NaN
SE-Lnn	0.796	Humid	CRO	0.252	SM	37.5	116.8	8.0	NaN	NaN
SN-Dhr	0.137	Arid	SAV	0.098	IL/MI	70.5	126.6	6.0	11.0	3.625
US-A32	0.451	Semi-arid	GRA	0.187	KA	22.7	944.5	0.0	NaN	NaN
US-AR1	0.298	Semi-arid	GRA	0.117	KA	46.7	911.0	0.0	NaN	NaN
US-AR2	0.303	Semi-arid	GRA	0.109	KA	66.9	940.7	0.0	NaN	NaN
US-ARb	0.440	Semi-arid	GRA	0.211	KA	28.7	763.2	3.0	NaN	NaN
US-ARc	0.440	Semi-arid	GRA	0.211	KA	30.1	762.0	0.0	NaN	NaN
US-ARM	0.492	Semi-arid	CRO	0.160	KA	20.6	907.7	13.0	NaN	NaN
US-Bi1	0.222	Semi-arid	CRO	0.199	IL/MI	26.5	81.5	1.0	NaN	NaN
US-Bi2	0.221	Semi-arid	CRO	0.238	IL/MI	25.0	76.3	0.0	NaN	NaN
US-Blo	0.897	Humid	ENF	0.155	KA	47.5	195.0	7.0	4.0	4.125
US-CF1	0.499	Semi-arid	CRO	0.232	KA	12.5	409.3	3.0	NaN	NaN
US-CF2	0.497	Semi-arid	CRO	0.237	KA	12.8	408.9	4.0	NaN	NaN
US-CF3	0.471	Semi-arid	CRO	0.210	KA	13.2	407.3	0.0	NaN	NaN
US-CF4	0.471	Semi-arid	CRO	0.198	KA	13.9	407.3	5.0	NaN	NaN
US-CRT	0.742	Humid	CRO	0.232	KA	31.0	903.5	0.0	NaN	NaN
US-CS2	0.789	Humid	ENF	0.225	IL/MI	72.8	1073.2	0.0	NaN	NaN
US-GLE	0.443	Semi-arid	ENF	0.105	IL/MI	47.0	1300.0	0.0	NaN	NaN
US-Goo	0.953	Humid	GRA	0.264	KA	13.1	429.9	1.0	NaN	NaN
US-Hn2	0.104	Arid	GRA	NaN	IL/MI	42.5	252.9	0.0	NaN	NaN
US-Hn3	0.104	Arid	OSH	0.044	IL/MI	42.3	253.2	NaN	NaN	NaN
US-HWB	0.911	Humid	CVM	0.270	IL/MI	25.1	268.1	0.0	NaN	NaN
US-IB2	0.815	Humid	GRA	0.241	KA	25.8	1243.3	0.0	NaN	NaN
US-Jo1	0.123	Arid	OSH	NaN	IL/MI	55.6	668.1	0.0	NaN	NaN
US-Jo2	0.128	Arid	OSH	0.036	IL/MI	54.0	671.0	15.0	5.5	4.000
US-KFS	0.688	Humid	GRA	0.224	KA	16.3	1133.2	0.0	NaN	NaN
US-KLS	0.449	Semi-arid	GRA	0.156	KA	26.2	1148.6	0.0	NaN	NaN
US-KS1	0.707	Humid	ENF	0.211	IL/MI	67.7	1.7	1.0	NaN	NaN
US-KS2	0.735	Humid	CSH	0.181	IL/MI	59.0	5.3	0.0	NaN	NaN
US-Lin	0.093	Arid	CRO	0.176	IL/MI	52.2	139.2	0.0	NaN	NaN

Continued on next page

Site	AI	Aridity	IGBP	NIRv	Mineral	Sand	dist. coast	TP	DpY	HpD
US-LWW	0.440	Semi-arid	GRA	0.184	IL/MI	36.9	710.1	0.0	NaN	NaN
US-Me1	0.460	Semi-arid	ENF	0.086	SM	42.3	223.1	0.0	NaN	NaN
US-Me2	0.705	Humid	ENF	0.131	IL/MI	52.3	231.6	6.0	NaN	NaN
US-Me3	0.404	Semi-arid	ENF	0.089	IL/MI	57.1	242.0	9.0	NaN	NaN
US-Me4	0.590	Dry subhumid	ENF	0.115	IL/MI	50.7	224.4	0.0	NaN	NaN
US-Me5	0.636	Dry subhumid	ENF	0.133	IL/MI	50.5	232.6	14.0	NaN	NaN
US-Me6	0.406	Semi-arid	ENF	0.087	IL/MI	57.4	241.3	3.0	NaN	NaN
US-MOz	0.739	Humid	DBF	0.315	KA	20.1	969.7	0.0	NaN	NaN
US-NC1	0.894	Humid	OSH	0.259	KA	46.4	14.0	15.0	NaN	NaN
US-NR1	0.431	Semi-arid	ENF	0.117	IL/MI	55.3	1197.3	0.0	NaN	NaN
US-Oho	0.761	Humid	DBF	0.296	KA	61.4	963.5	0.0	NaN	NaN
US-ONA	0.165	Arid	GRA	0.048	IL/MI	42.7	869.4	0.0	NaN	NaN
US-Rms	0.353	Semi-arid	CSH	0.114	SM	45.5	692.9	0.0	NaN	NaN
US-Ro1	0.701	Humid	CRO	0.240	KA	46.1	1345.1	2.0	NaN	NaN
US-Ro4	0.709	Humid	GRA	0.263	KA	46.9	1371.9	10.0	NaN	NaN
US-Ro5	0.707	Humid	CRO	0.268	KA	45.9	1370.1	0.0	NaN	NaN
US-Ro6	0.709	Humid	CRO	0.268	KA	45.4	1363.8	0.0	NaN	NaN
US-Rws	0.208	Semi-arid	OSH	0.045	SM	35.9	687.6	21.0	13.0	5.250
US-Snf	0.198	Arid	GRA	0.209	IL/MI	28.7	43.9	0.0	NaN	NaN
US-SRC	0.157	Arid	OSH	0.046	IL/MI	44.1	277.1	17.0	6.0	6.000
US-SRG	0.223	Semi-arid	GRA	0.087	IL/MI	42.5	274.0	1.0	NaN	NaN
US-SRM	0.184	Arid	WSA	0.068	IL/MI	43.0	254.7	8.0	NaN	NaN
US-Sta	0.210	Semi-arid	OSH	0.048	IL/MI	47.0	1277.9	0.0	NaN	NaN
US-Syv	0.956	Humid	MF	0.225	IL/MI	55.4	905.1	8.0	NaN	NaN
US-Ton	0.344	Semi-arid	WSA	0.143	IL/MI	40.8	136.6	39.0	24.5	4.500
US-Tw2	0.217	Semi-arid	CRO	0.204	IL/MI	28.9	69.3	3.0	NaN	NaN
US-Tw3	0.217	Semi-arid	CRO	0.205	IL/MI	26.8	70.1	0.0	NaN	NaN
US-UMd	0.816	Humid	DBF	0.231	IL/MI	58.2	720.3	0.0	NaN	NaN
US-Var	0.335	Semi-arid	GRA	0.165	IL/MI	40.5	136.0	46.0	80.0	5.000
US-WCr	0.924	Humid	DBF	0.356	IL/MI	49.6	974.5	9.0	NaN	NaN
US-Whs	0.150	Arid	OSH	0.043	IL/MI	60.7	332.7	0.0	NaN	NaN
US-Wkg	0.168	Arid	GRA	0.060	IL/MI	58.7	347.4	0.0	NaN	NaN
US-xAE	0.355	Semi-arid	GRA	0.102	KA	40.4	801.7	1.0	NaN	NaN
US-xBR	1.279	Humid	DBF	0.323	IL/MI	54.8	100.8	0.0	NaN	NaN
US-xCL	0.471	Semi-arid	GRA	0.192	KA	44.4	526.8	0.0	NaN	NaN
US-xCP	0.213	Semi-arid	GRA	0.064	KA	54.2	1306.5	3.0	NaN	NaN
US-xDC	0.379	Semi-arid	GRA	0.217	KA	37.5	1166.1	4.0	NaN	NaN
US-xDL	0.910	Humid	MF	0.271	KA	32.8	202.8	1.0	NaN	NaN
US-xDS	0.682	Humid	CVM	0.167	IL/MI	78.9	74.2	NaN	NaN	NaN
US-xGR	1.071	Humid	DBF	0.336	KA	35.4	442.4	0.0	NaN	NaN
US-xHA	1.098	Humid	DBF	0.326	IL/MI	60.3	102.4	NaN	NaN	NaN
US-xJE	0.827	Humid	ENF	0.221	IL/MI	76.8	116.0	15.0	28.0	4.250
US-xKA	0.570	Dry subhumid	GRA	0.193	KA	17.2	1216.9	0.0	NaN	NaN
US-xKZ	0.577	Dry subhumid	GRA	0.244	KA	13.4	1213.2	0.0	NaN	NaN

Continued on next page

Site	AI	Aridity	IGBP	NIRv	Mineral	Sand	dist. coast	TP	DpY	HpD
US-xMB	0.175	Arid	OSH	0.039	IL/MI	56.3	861.4	2.0	NaN	NaN
US-xML	1.044	Humid	DBF	0.358	KA	44.6	423.4	0.0	NaN	NaN
US-xNG	0.337	Semi-arid	GRA	0.168	IL/MI	43.7	1256.5	2.0	NaN	NaN
US-xNQ	0.165	Arid	OSH	0.048	IL/MI	42.7	869.4	NaN	NaN	NaN
US-xRM	0.371	Semi-arid	ENF	0.105	IL/MI	58.3	1216.3	NaN	NaN	NaN
US-xSB	0.796	Humid	ENF	0.148	IL/MI	85.4	68.6	14.0	NaN	NaN
US-xSE	0.813	Humid	DBF	0.290	KA	43.5	21.1	1.0	NaN	NaN
US-xSR	0.155	Arid	OSH	0.046	IL/MI	44.5	262.7	9.0	NaN	NaN
US-xST	0.889	Humid	DBF	0.277	IL/MI	54.4	982.1	9.0	NaN	NaN
US-xTA	0.980	Humid	ENF	0.240	KA	48.1	252.6	7.0	NaN	NaN
US-xTR	0.887	Humid	DBF	0.281	IL/MI	56.8	983.5	4.0	NaN	NaN
US-xUK	0.684	Humid	DBF	0.278	KA	19.0	1131.9	0.0	NaN	NaN
US-xUN	0.953	Humid	MF	0.255	IL/MI	55.0	912.9	3.0	NaN	NaN
US-xYE	0.478	Semi-arid	ENF	0.107	KA	44.6	1387.8	NaN	NaN	NaN
ZM-Mon	0.398	Semi-arid	WSA	0.170	IL/MI	84.0	1206.9	0.0	NaN	NaN

References

Hohenbrink, T. L., Jackisch, C., Durner, W., Germer, K., Iden, S. C., Kreiselmeier, J., Leuther, F., Metzger, J. C., Naseri, M., and Peters, A. (2023). Soil hydraulic characteristics in a wide range of saturation and soil properties. [Dataset] https://dataservices.gfz-potsdam.de/panmetaworks/showshort.php?id=9a051f59-b3a2-11ed-95b8-f851ad6d1e4b (last access: 24.09.2024).

Thomas, D., Middleton, N., and UNEP (1997). World atlas of desertification. United Nation Environment Programme (UNEP) Publication, UNEP(017)/W927, London ; New York ; Sydney ; Auckland : Arnold : UNEP.
